# Observed Clinical and Operational Factors Are Associated with Biomarker Test Utilization in Respiratory Virus RT-PCR Testing: A Real-World Analysis of Inflammatory, Cardiac, and Thrombotic Biomarkers

**DOI:** 10.3390/diagnostics16193232

**Published:** 2026-10-07

**Authors:** Yonghee Lee, Ga-Yeon Kim, Jae Kyung Kim

**Affiliations:** 1 Department of Biomedical Laboratory Science, College of Health Sciences, Dankook University, 119 Dandae-ro, Dongnam-gu, Cheonan-si 31116, Republic of Korea; ghwn874@naver.com; 2 Department of Laboratory Medicine, Kyung Hee University Hospital, 23, Kyungheedae-ro, Dongdaemun-gu, Seoul-si 02447, Republic of Korea; 3 Department of Public Health, Graduate School, Dankook University, 119 Dandae-ro, Dongnam-gu, Cheonan-si 31116, Republic of Korea; sysnhj77@gmail.com

**Keywords:** respiratory viruses, RT-PCR, diagnostic stewardship, laboratory test utilization, biomarker measurement, CRP, procalcitonin, cardiac biomarkers, D-dimer, selection bias

## Abstract

**Background/Objectives:** Routinely collected biomarker data are generated through selective testing. We examined associations of observed clinical and operational factors with biomarker availability and explored the incremental discrimination provided by biomarker concentrations for respiratory viral RT-PCR positivity. **Methods:** This retrospective study included 7726 testing episodes for 13 respiratory viral targets from July 2020 to March 2025. Seven biomarkers were linked using a ±24-h reporting-time window. Models included age, sex, requesting department, operational time, calendar month, and year. Additional analyses included inverse probability weighting (IPW), weight and overlap diagnostics, and five repetitions of five-fold cross-validation. **Results:** Viral positivity was 12.72%. Biomarker availability ranged from 98.87% for C-reactive protein to 22.87% for B-type natriuretic peptide. Procalcitonin (PCT) result availability was associated with lower odds of viral positivity after adjustment (OR, 0.702; 95% CI, 0.583–0.846). Among episodes with a PCT result, the concentration association was close to the null (OR per doubling, 0.991; 95% CI, 0.953–1.030). The complete-cohort base model had an apparent area under the receiver operating characteristic curve (AUC) of 0.8263. The largest apparent ΔAUC was 0.00418 for D-dimer; its mean out-of-fold ΔAUC was 0.00367. IPW diagnostics showed substantial reductions in effective sample size and sensitivity to extreme weights for some biomarkers. **Conclusions:** Biomarker availability was associated with the observed clinical and operational factors, whereas concentrations added little discrimination for positivity within the available viral panel. These findings illustrate selection in retrospective laboratory data and do not assess biomarker usefulness for their intended clinical indications.

## 1. Introduction

Respiratory viral infections remain a major clinical burden across the lifespan, with circulation varying by age, season, and calendar period; these patterns were substantially disrupted during and after the coronavirus disease 2019 pandemic [1,2,3,4,5,6]. Multiplex respiratory-virus RT-PCR has improved etiologic detection, but a positive or negative molecular result does not by itself characterize host response or systemic complications [7].

Inflammatory biomarkers are therefore frequently used as adjuncts. C-reactive protein (CRP) reflects a broad acute-phase response, whereas procalcitonin (PCT) is used primarily to support the assessment of bacterial infection and antimicrobial stewardship; neither marker is an etiologic classifier for respiratory viral infection [8,9,10].

Cardiac and thrombotic biomarkers may also be measured when respiratory illness raises concern for myocardial injury, heart failure, or thromboembolism. Acute respiratory infection can precipitate cardiovascular events, whereas the use and interpretation of troponin, B-type natriuretic peptide (BNP), N-terminal pro-B-type natriuretic peptide (NT-proBNP), and D-dimer depend strongly on their underlying clinical indications and patient characteristics [11,12,13,14,15,16,17].

A limitation of retrospective laboratory research is that these measurements are not generated at random. The presence of a result depends on patient characteristics, the requesting department, timing, and the clinical question that prompted testing. Treating unavailable measurements as ordinary missing data can therefore bias estimated associations [18,19]. Diagnostic stewardship likewise considers appropriate test selection, timing, and interpretation in addition to analytical performance [20].

Our previous study used the overlapping CRP-available respiratory-virus archive to describe CRP distributions and respiratory viral detection patterns [21]. Here, we address a separate methodological question: how biomarker availability is associated with observed clinical and operational factors and how much incremental discrimination measured concentrations provide after adjustment for those factors.

The primary objective was to characterize the availability of seven inflammatory, cardiac, and thrombotic biomarkers and its associations with age, sex, requesting department, operational time, and calendar period. Secondary objectives were to compare measured and unmeasured episodes, explore the incremental discrimination of concentrations, and examine observed selection using IPW. Respiratory viral RT-PCR positivity was used as a common recorded outcome for these comparisons, rather than as an intended diagnostic target of the cardiac and thrombotic biomarkers. The RT-PCR-negative group was not assumed to represent a single disease category, and the analysis was not designed to establish biological effects of viral infection.

## 2. Materials and Methods

### 2.1. Study Design and Population

This single-center retrospective observational study was conducted at Kyung Hee University Hospital, Seoul, Republic of Korea. The source cohort consisted of respiratory viral RT-PCR testing episodes performed between 1 July 2020 and 30 March 2025. Outpatient, emergency, and inpatient testing episodes were eligible if the respiratory viral RT-PCR result, age, sex, laboratory accession timestamp, and requesting-department code were available.

All eligible records during the study period were included; no a priori sample-size calculation was performed. The final source cohort comprised 7726 RT-PCR testing records. For this analysis, an episode was operationally defined as a row in the released dataset containing an episode-level accession timestamp, requesting-department code, demographic variables, and reported viral results. An episode was not assumed to represent a unique patient or clinical encounter. Patient, encounter, and specimen identifiers were unavailable; therefore, the number of unique patients, repeated episodes within patients, and repeat measurements from the same encounter or specimen could not be established. The primary analyses retained all source records, and exact-record matching was used only to identify candidates for a separate sensitivity analysis.

Following the IRB exemption determination and informed-consent waiver, the hospital medical information management team extracted records using the approved eligibility criteria and a ±24-h window around the RT-PCR reporting time for biomarker linkage. According to the extraction specification, the result closest in time was retained when multiple values were available. Episodes without a linked result for a given biomarker remained in the source cohort but were excluded from its concentration analyses. The released dataset contained an RT-PCR accession timestamp but no individual biomarker ordering, collection, or reporting timestamps. The linkage could not be independently reconstructed, and narrower-window or before-versus-after analyses could not be performed.

The window was treated as an operational linkage criterion rather than as evidence that the measurements arose from the same encounter or clinical decision. Reporting-time linkage may include measurements obtained for different indications and does not establish temporal ordering.

Reporting was guided by the Strengthening the Reporting of Observational Studies in Epidemiology statement and the REporting of studies Conducted using Observational Routinely-collected health Data statement [22,23].

### 2.2. Respiratory Viral RT-PCR Testing

Nasopharyngeal swab specimens were collected in ASAN Transport Medium (Asan Pharm, Seoul, Republic of Korea), stored at 4 °C, and tested within 24 h after collection. Nucleic acids were extracted using the AdvanSure E3 System and AdvanSure Nucleic Acid R Kit (LG Chem, Seoul, Republic of Korea). Reverse transcription, amplification, and detection were performed using the AdvanSure RV-Plus real-time RT-PCR kit and SLAN-96P Real-Time PCR System (LG Chem, Seoul, Republic of Korea).

For each specimen, four reaction tubes containing 5 μL of predispensed primer/probe mixture were used. Each reaction consisted of 10 μL of RT-PCR premix and 5 μL of extracted nucleic acid, for a final reaction volume of 20 μL. Reverse transcription was performed at 50 °C for 10 min, followed by initial denaturation/activation at 95 °C for 30 s; 10 cycles at 95 °C for 15 s, 53 °C for 30 s, and 60 °C for 30 s; and an additional 30 cycles under the same conditions. Fluorescence was measured during the 60 °C step.

The assay detects 15 respiratory viral targets. Human metapneumovirus and enterovirus results were not included in the dataset available under the current Institutional Review Board and data-use approval. The analytical dataset therefore comprised 13 targets: adenovirus; RSV A and B; influenza virus A and B; parainfluenza virus types 1, 2, and 3; rhinovirus; human coronaviruses 229E, NL63, and OC43; and bocavirus. SARS-CoV-2 was not included in this panel. The numbers of unperformed tests and missing results for these targets could not be determined, and their absence was not treated as a negative result.

A cycle-threshold (Ct) value < 27 was interpreted as positive. This threshold corresponded to the assay-specific interpretive criterion used in the routine clinical laboratory standard operating procedure throughout the study period and was not modified for this analysis. Positive, negative, and internal controls were included in each run according to the manufacturer’s instructions. The analysis used the categorical positive and negative results supplied in the extracted laboratory records. Raw Ct values and longitudinal records of assay or protocol changes were not available in the released dataset, so target- and time-specific analytical comparability could not be independently assessed from these records.

Respiratory viral positivity was defined as detection of at least one of the 13 included viral targets. Testing episodes were additionally classified as negative, single viral detection, dual viral detections, or three or more detections for descriptive analyses. For selected adjusted analyses, dual and ≥3 detections were combined as multiple detections because of sparse biomarker availability in the latter categories.

### 2.3. Biomarker Measurements

Seven biomarkers were analyzed: CRP, PCT, CK-MB, hs-cTnI, BNP, D-dimer, and NT-proBNP. Measurements were obtained during routine clinical care without a study-directed testing protocol. The standardized biomarker labels and units were CRP (mg/dL), PCT (ng/mL), CK-MB (ng/mL), hs-cTnI (pg/mL), BNP (pg/mL), D-dimer (μg/mL FEU), and NT-proBNP (pg/mL). FEU denotes fibrinogen-equivalent units. Biomarker availability was defined as the presence of a linked result in the extracted record. Absence of a linked result did not establish that the test had not been ordered or performed.

CRP was measured using CRP Latex Reagent on an AU5800 analyzer (Beckman Coulter, Brea, CA, USA). PCT was measured using Elecsys BRAHMS PCT reagent on a Cobas e 801 analyzer (Roche Diagnostics, Mannheim, Germany).

CK-MB was measured in serum using the Access CK-MB assay on the UniCel DxI 800 Access Immunoassay System (Beckman Coulter); the reportable range was 0.1–300 ng/mL. hs-cTnI was measured in serum using the Access hsTnI assay on the same platform, with a reportable range of 2.3–27,027 pg/mL. BNP was measured in EDTA plasma using the Access BNP assay, with a reportable range of 3–5000 pg/mL. D-dimer was measured in sodium-citrate plasma using STA-Liatest D-Di Plus reagent on the STA R Max automated coagulation analyzer (Diagnostica Stago, Asnières-sur-Seine, France), with a reportable range of 0.27–20.0 µg/mL fibrinogen-equivalent units. NT-proBNP was measured in serum using Elecsys proBNP II reagent on the Cobas e 801 analyzer; its direct reportable range was 5–35,000 pg/mL and could be extended to 70,000 pg/mL using the manufacturer’s two-fold dilution procedure.

Longitudinal records of analyzer, reagent, calibration, and laboratory-procedure changes were not included in the released dataset; analytical comparability across the study period therefore could not be independently assessed from these data.

Values explicitly reported with a “>” sign were assigned the corresponding reported upper limit, and values explicitly reported with a “<” sign were assigned one-half of the reported lower limit. Unflagged numeric values were retained without additional threshold-based recoding. The numbers and proportions of explicitly flagged values were summarized separately for each biomarker, using measured episodes as the denominator. Concentrations were analyzed continuously rather than dichotomized at clinical decision thresholds.

### 2.4. Observed Clinical and Operational Variables

Age, sex, requesting department, operational time, calendar month, and calendar year were the available adjustment variables. These variables did not capture the full clinical decision-making process or the indications for biomarker ordering. Age was modeled continuously in the primary models, with effects expressed per 10-year increase. The categories < 5, 5–18, 19–60, and >60 years were retained for descriptive analyses. Calendar month and year were entered as categorical variables, with January and 2020 as reference categories. Using monthly categories avoided imposing a single seasonal waveform; a sine/cosine specification and flexible age modeling were examined in additional sensitivity analyses.

#### 2.4.1. Requesting Department

The raw dataset contained 26 requesting-department codes. Six major departments were retained as individual groups: Pulmonology (IP), Hematology (IH), Pediatrics (PE), Nephrology (IN), Cardiology (IC), and Infectious Diseases (II).

Gastroenterology (IG), Rheumatology (IR), and Endocrinology (IE) were combined as Other Internal Medicine.

All remaining departments were combined as Other Departments: Neurology (NR), Korean Medicine Hospital consultation (NH), Orthopedic Surgery (OS), Neurosurgery (NS), General Surgery (GS), Urology (UR), Rehabilitation Medicine (PM), Psychiatry (NP), Emergency Medicine (ED), Otorhinolaryngology (EN), Thoracic Surgery (TS), Obstetrics and Gynecology (OB), Health Screening (DC), Plastic Surgery (PS), Family Medicine (FM), Dermatology (DE), and Ophthalmology (OP).

The final department variable therefore contained eight categories. Pulmonology was used as the reference department.

Only 12 episodes carried the Emergency Medicine requesting-department code, including three virus-positive episodes. This code could not be assumed to identify all episodes managed in an emergency department. A separate adjusted emergency-department analysis was therefore not performed.

#### 2.4.2. Operational Time

Operational time was derived from the laboratory accession timestamp of each RT-PCR testing episode.

Sundays, official national public holidays, and substitute holidays in the Republic of Korea were classified as holidays, regardless of time.

On non-holiday dates, routine operating hours were defined according to the operating schedule of Kyung Hee University Medical Center as 08:00 to <17:00 Monday through Friday and 08:00 to <12:00 on Saturday.

All remaining periods were classified as after-hours, including weekday evening/nighttime and early-morning periods and Saturday from 12:00 onward.

Operational time was presented descriptively as routine hours, after-hours, and holidays. The main adjusted analyses used routine hours versus after-hours/holiday.

The timestamp corresponded to RT-PCR laboratory accession rather than the exact ordering or collection time for each biomarker. Operational time was therefore interpreted as the temporal context of the testing episode.

### 2.5. Statistical Analysis

Categorical variables are presented as counts and percentages, and continuous variables as medians and interquartile ranges (IQRs). Viral positivity across operational-time categories was compared using Pearson’s chi-square test.

The discrimination analyses using the joint CRP/PCT complete-case subset entered CRP and PCT as untransformed concentrations. In the biomarker-specific concentration analyses and concentration-based sensitivity analyses, biomarker concentrations were log2-transformed. General linear models of concentrations across viral-detection groups were adjusted for age, sex, and calendar month, with coefficients back-transformed to adjusted geometric mean ratios. For the three viral-detection categories (negative, single detection, and multiple detections), Bonferroni adjustment covered all three pairwise comparisons within each biomarker. Both the two-sided *p* values and confidence intervals for these comparisons were adjusted; confidence limits on the log2 scale were back-transformed to geometric mean ratios. Estimates were back-transformed before rounding. These pairwise results were interpreted following a significant overall group effect.

Separate logistic regression models evaluated measurement of PCT, CK-MB, hs-cTnI, BNP, D-dimer, and NT-proBNP using the observed clinical and operational variables. CRP measurement status was described but not modeled because 98.87% of episodes had a CRP result and some departments had no unmeasured episodes, raising separation concerns; CRP-weighted estimates are therefore not reported. CRP concentration was retained in the discrimination analyses.

Separate logistic models evaluated the association between each biomarker’s measurement status and respiratory viral positivity. The reduced models included age, sex, and calendar month. Full models additionally included requesting department, operational time, and calendar year. Measurement-status associations were distinguished from the associations of biomarker concentrations among measured episodes.

Within each biomarker-measured subset, a base logistic model included age, sex, requesting department, operational time, calendar month, and calendar year. An extended model added the corresponding log2-transformed biomarker concentration while retaining the same observations. The base and extended models contained 26 and 27 slope parameters, respectively, excluding the intercept; biomarker ORs represented associations per two-fold increase in concentration. Positive-event counts, events per parameter, design-matrix rank, and numerical stability were examined.

Univariate AUCs were calculated using biomarker concentrations as ranking scores, with higher concentrations assigned to the positive direction. Model-based ROC curves were obtained from predicted probabilities of viral positivity. Apparent AUC and the descriptive increment ΔAUC = AUC(extended model) − AUC(base model) were calculated using unrounded values. These analyses examined incremental discrimination and were not intended to establish clinical predictive utility.

Internal validation used five repetitions of stratified five-fold cross-validation within each biomarker-measured subset. Base and extended models used identical splits in each repetition, and records identical across all 24 source fields were kept in the same fold. Models were fitted without penalization within each training fold, and held-out predictions were pooled to calculate one out-of-fold AUC per model and repetition. Mean AUCs and mean ΔAUCs were summarized across repetitions using random seeds 20260921–20260925. Patient-level separation could not be ensured because patient identifiers were unavailable. Ranges across repetitions were reported as descriptive stability measures, not confidence intervals.

Additional post hoc sensitivity analyses used restricted cubic splines for age, with four knots at the full-cohort 5th, 35th, 65th, and 95th percentiles (1, 65, 77, and 89 years). Nonlinearity was assessed by likelihood-ratio comparison with the corresponding linear model. Biomarker nonlinearity was assessed using four knots at the same percentiles of log2 concentration within each measured subset, with spline-adjusted age. Calendar month was alternatively represented by sine and cosine terms for a 12-month cycle. Further analyses excluded exact-record duplicate candidates or values explicitly flagged as below or above measurement limits.

Categorical-age concentration models were also examined, but sparse combinations of age and department produced unstable nuisance-parameter estimation in some subsets, including rank deficiency in the BNP subset. Those models were not used as confirmatory evidence of robustness; spline-based age sensitivity results are presented in Supplementary Table S3.

As a secondary analysis, inverse probability weighting (IPW) was used to evaluate selection associated with biomarker measurement [24]. The probability of measurement was modeled using age, sex, requesting department, operational time, calendar month, and calendar year. Among measured episodes, stabilized inverse probability weights were calculated as the marginal probability of measurement divided by the estimated conditional probability of measurement. Estimated measurement probabilities were restricted to 0.01–0.99, and truncation of weights at the 1st and 99th percentiles was evaluated as a sensitivity analysis. Effective sample size was calculated as (Σw)^2^/Σw^2^ to evaluate weighting stability.

Diagnostics included the distribution of stabilized weights, the proportion of total weight carried by the highest-weight 1% of measured episodes, and the ratio of effective sample size to measured sample size. Estimated measurement-probability distributions were compared between measured and unmeasured episodes before probability clipping, including the number of episodes with estimated probabilities below 0.01. Weight-capping cutoffs used linearly interpolated empirical 1st and 99th percentiles. These diagnostics assessed support and sensitivity to weighting conditional on the observed variables and were not intended to correct for unmeasured clinical indications.

Statistical analyses were performed using IBM SPSS Statistics version 29.0 (IBM Corp., Armonk, NY, USA) and Python version 3.10 (Python Software Foundation, Wilmington, DE, USA); figures were prepared using Matplotlib version 3.10.8. ChatGPT (OpenAI; 6.0 Astra) assisted in drafting the Python scripts. Tests were two-sided. Bonferroni adjustment was applied to the specified post hoc group comparisons. Biomarker-specific regression analyses and additional sensitivity analyses were exploratory, and their *p* values were not adjusted for multiple testing. *p* values were interpreted alongside effect sizes, confidence intervals, numerical stability, and discrimination metrics.

### 2.6. Ethical Considerations

The study was conducted in accordance with the Declaration of Helsinki. The Institutional Review Board of Kyung Hee University Hospital determined that the study was exempt from review (IRB File No. KHUH IRB 2025-09-027; determination date: 15 September 2025).

The requirement for individual informed consent was waived because of the retrospective nature of the study. We used previously collected, de-identified medical record and clinical laboratory data and the study involved no additional intervention or specimen collection.

## 3. Results

### 3.1. Cohort Characteristics

The study included 7726 respiratory viral RT-PCR testing episodes. Median age was 72 years (IQR, 58–80 years), and 4643 episodes (60.10%) were from male patients. Respiratory viruses were detected in 983 episodes (12.72%), whereas 6743 (87.28%) were negative. Single detection occurred in 854 episodes (11.05%), dual detection in 112 (1.45%), and three or more detections in 17 (0.22%). Baseline demographic, virological, and biomarker characteristics are summarized in Table 1.

Four pairs of records were identical across all 24 available fields, including the episode timestamp. These were considered duplicate candidates rather than confirmed duplicate specimens or encounters. Excluding the second record in each pair yielded 7722 episodes, including 982 positive episodes. The primary analysis retained the source cohort, with exclusion of duplicate candidates examined only in sensitivity analyses.

Age and calendar period were retained as contextual covariates in subsequent models. Detailed viral patterns by age, season, and co-detection in the overlapping CRP-available archive have been reported previously [21] and were not treated as primary findings of the present analysis.

### 3.2. Selective Biomarker Measurement

Biomarker availability varied substantially. CRP was measured in 7639 episodes (98.87%), PCT in 5858 (75.82%), CK-MB in 4608 (59.64%), hs-cTnI in 4672 (60.47%), BNP in 1767 (22.87%), D-dimer in 4787 (61.96%), and NT-proBNP in 2441 (31.59%). Biomarker availability and the characteristics of measured and unmeasured testing episodes are summarized in Table 2.

**Figure 1:**
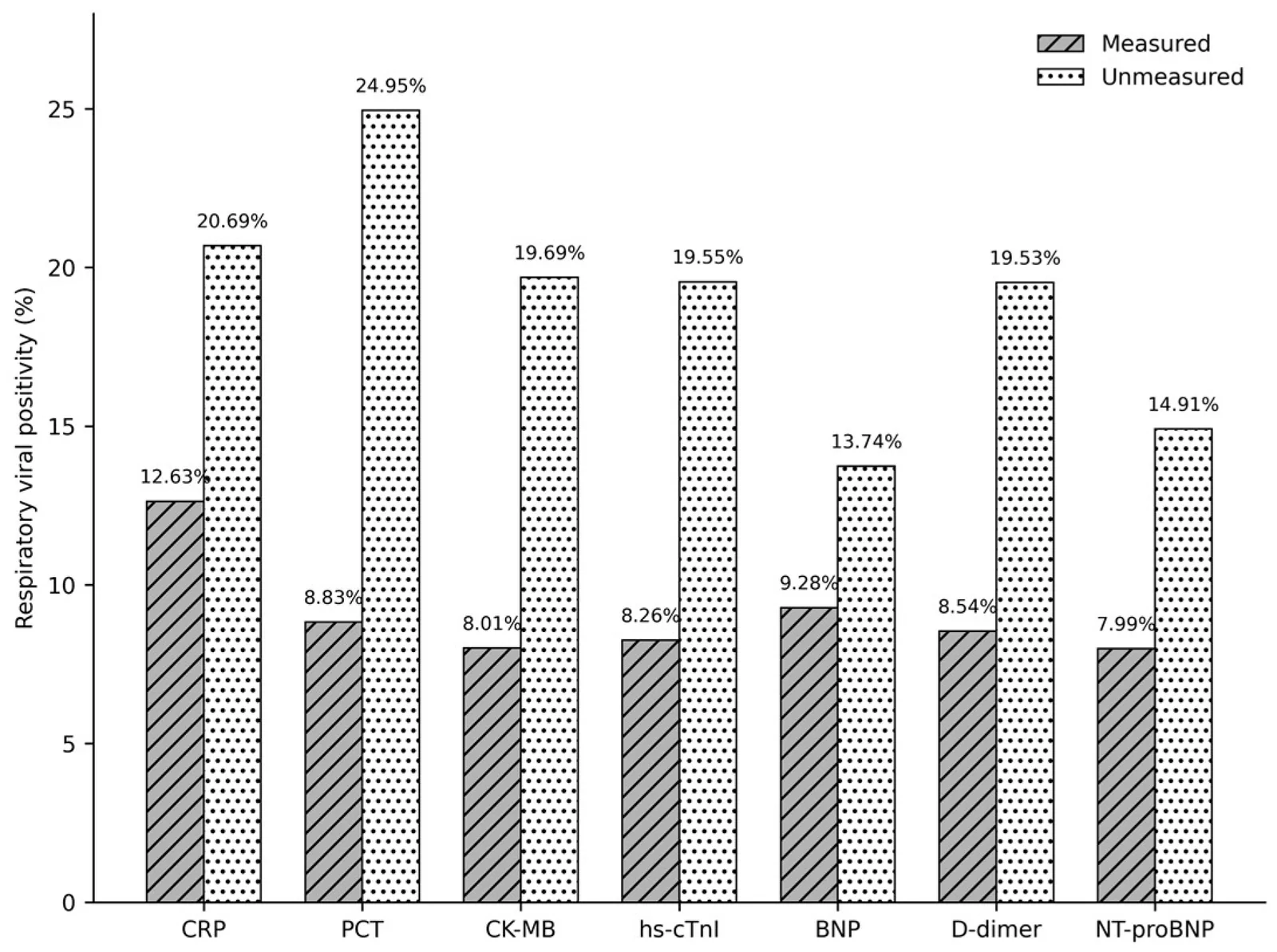
Respiratory viral positivity according to biomarker measurement status. Viral positivity is shown separately for testing episodes in which each biomarker was measured and unmeasured. Abbreviations: CRP, C-reactive protein; PCT, procalcitonin; CK-MB, creatine kinase-MB; hs-cTnI, high-sensitivity cardiac troponin I; BNP, B-type natriuretic peptide; NT-proBNP, N-terminal pro-B-type natriuretic peptide.

For every biomarker, measured episodes had a higher median age than unmeasured episodes, and viral positivity was lower among measured episodes. The largest difference occurred for PCT: positivity was 8.83% in measured episodes versus 24.95% in unmeasured episodes. For CK-MB, hs-cTnI, BNP, D-dimer, and NT-proBNP, measured-versus-unmeasured positivity was 8.01% versus 19.69%, 8.26% versus 19.55%, 9.28% versus 13.74%, 8.54% versus 19.53%, and 7.99% versus 14.91%, respectively (Figure 1).

PCT-measured episodes had a higher median age than PCT-unmeasured episodes (median 74 vs. 62 years) and had higher CRP (10.18 vs. 3.65 mg/dL). In a model adjusted for age, sex, and calendar month, PCT measurement was associated with lower odds of viral positivity (OR, 0.571; 95% CI, 0.485–0.674).

After requesting department, operational time, and calendar year were added to the age-, sex-, and month-adjusted model, the association for PCT measurement status was attenuated but remained below the null (adjusted OR, 0.702; 95% CI, 0.583–0.846; *p* < 0.001). D-dimer measurement status showed a weaker association (OR, 0.819; 95% CI, 0.672–0.999; *p* = 0.049), whereas the confidence intervals for the other measurement-status associations included the null. These measurement-status analyses are distinct from analyses of biomarker concentration. The fully adjusted measurement-status associations are summarized in Supplementary Table S8.

### 3.3. Inflammatory Biomarker Concentrations and Discrimination

In the joint CRP/PCT complete-case subset of 5849 episodes, the univariate AUCs were 0.5029 for CRP and 0.4898 for PCT. The age-, sex-, and month-adjusted base-model AUC was 0.6758; adding untransformed CRP, untransformed PCT, or both yielded AUCs of 0.6787, 0.6760, and 0.6797, respectively. These analyses are summarized in Table 3.

### 3.4. Cardiac and Thrombotic Biomarkers Across Viral Detection Groups

After adjustment for age, sex, and calendar month, D-dimer was the only biomarker showing a significant overall difference across viral-detection categories. The adjusted geometric mean ratio for single viral detection versus a negative RT-PCR result was 0.814 (Bonferroni-adjusted 95% CI, 0.717–0.925; Bonferroni-adjusted *p* < 0.001), whereas the multiple-detection group did not differ from the negative group. CK-MB, hs-cTnI, BNP, and NT-proBNP showed no significant adjusted group effects. The distribution of D-dimer according to viral detection category is shown in Supplementary Figure S1.

The univariate AUCs were 0.5057 for CK-MB, 0.4897 for hs-cTnI, 0.4777 for BNP, 0.4423 for D-dimer, and 0.5128 for NT-proBNP. In age-, sex-, and month-adjusted models, D-dimer was associated with lower odds of viral positivity per two-fold increase (OR, 0.872; 95% CI, 0.812–0.938; *p* < 0.001). These findings are summarized in Table 4.

### 3.5. Requesting Department and Biomarker Utilization

Biomarker measurement varied markedly according to requesting department. Pediatrics had the highest respiratory viral positivity (49.2%) but the lowest measurement rates for most cardiac and thrombotic biomarkers. CK-MB was measured in only 2.3% of Pediatric episodes, compared with 87.1% of Cardiology episodes, whereas hs-cTnI ranged from 9.6% in Pediatrics to 86.9% in Cardiology. NT-proBNP measurement ranged from 9.2% in Pediatrics to 60.5% in Cardiology.

**Figure 2:**
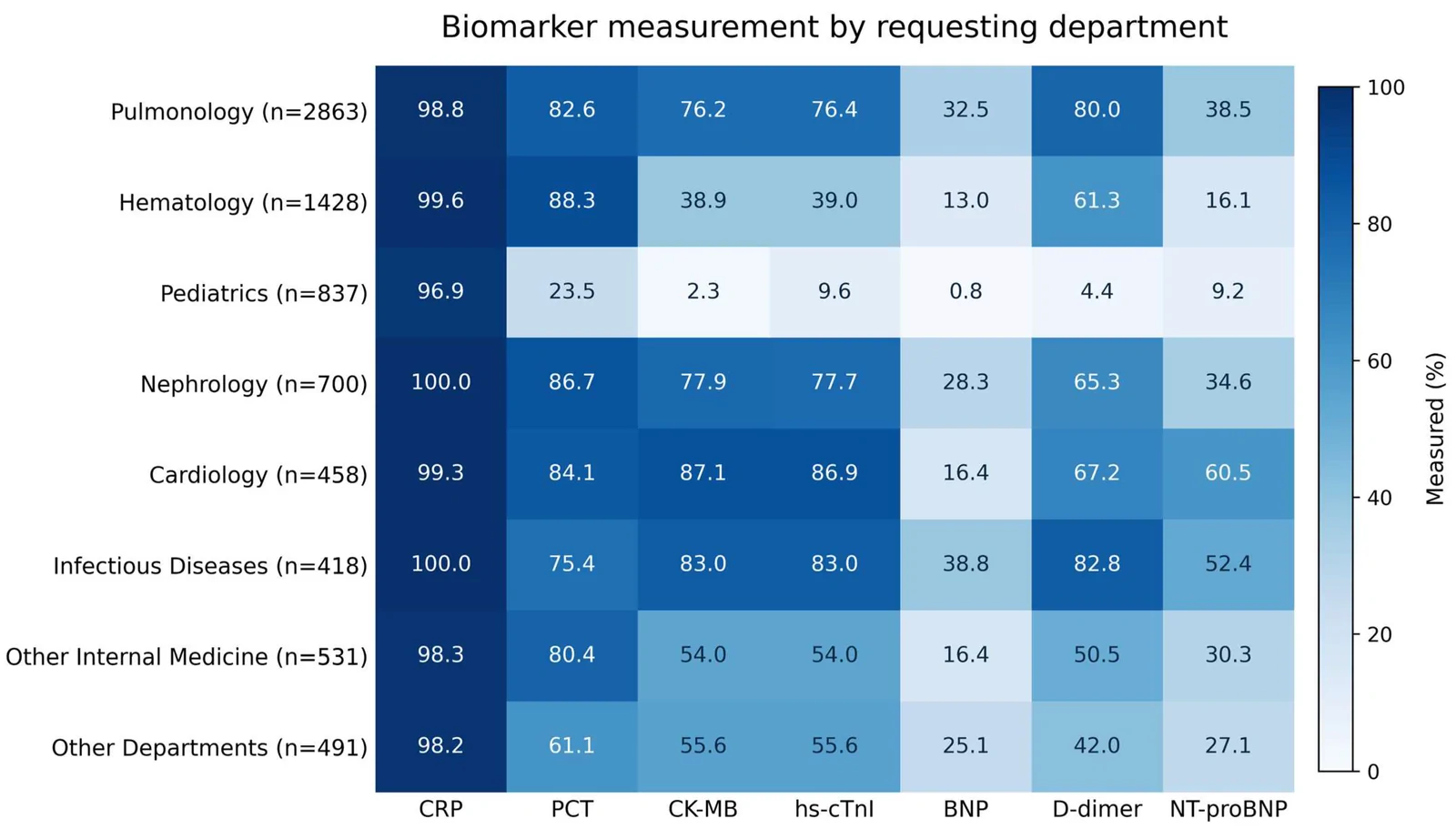
Biomarker measurement rates by requesting department. Each cell shows the percentage of episodes with a measured result within the corresponding department; department-specific episode counts are shown in the row labels. Color indicates measurement rate from 0% to 100%. Abbreviations: CRP, C-reactive protein; PCT, procalcitonin; CK-MB, creatine kinase-MB; hs-cTnI, high-sensitivity cardiac troponin I; BNP, B-type natriuretic peptide; NT-proBNP, N-terminal pro-B-type natriuretic peptide.

D-dimer measurement was highest in Infectious Diseases (82.8%) and Pulmonology (80.0%) and lowest in Pediatrics (4.4%). In contrast, CRP was measured in ≥96.9% of episodes across all department categories. Department-specific biomarker measurement rates are presented in Table 5 and visualized in Figure 2.

### 3.6. Operational Time and Selective Measurement

Among 7726 testing episodes, 6685 (86.53%) were accessioned during routine hours, 150 (1.94%) during non-holiday after-hours, and 891 (11.53%) on Sundays or Korean public holidays. Sunday and public-holiday accessions accounted for 891 of the 1041 episodes (85.6%) in the combined after-hours/holiday category. Detailed utilization according to operational time is presented in Supplementary Table S1.

Respiratory viral positivity did not differ substantially among routine hours, after-hours, and holidays (12.63%, 12.67%, and 13.47%, respectively; *p* = 0.778). In contrast, several biomarker measurement rates were higher outside routine hours. PCT measurement increased from 75.15% during routine hours to 81.48% on holidays, CK-MB from 58.67% to 65.88%, hs-cTnI from 59.52% to 66.67%, and D-dimer from 61.08% to 68.01%.

**Figure 3:**
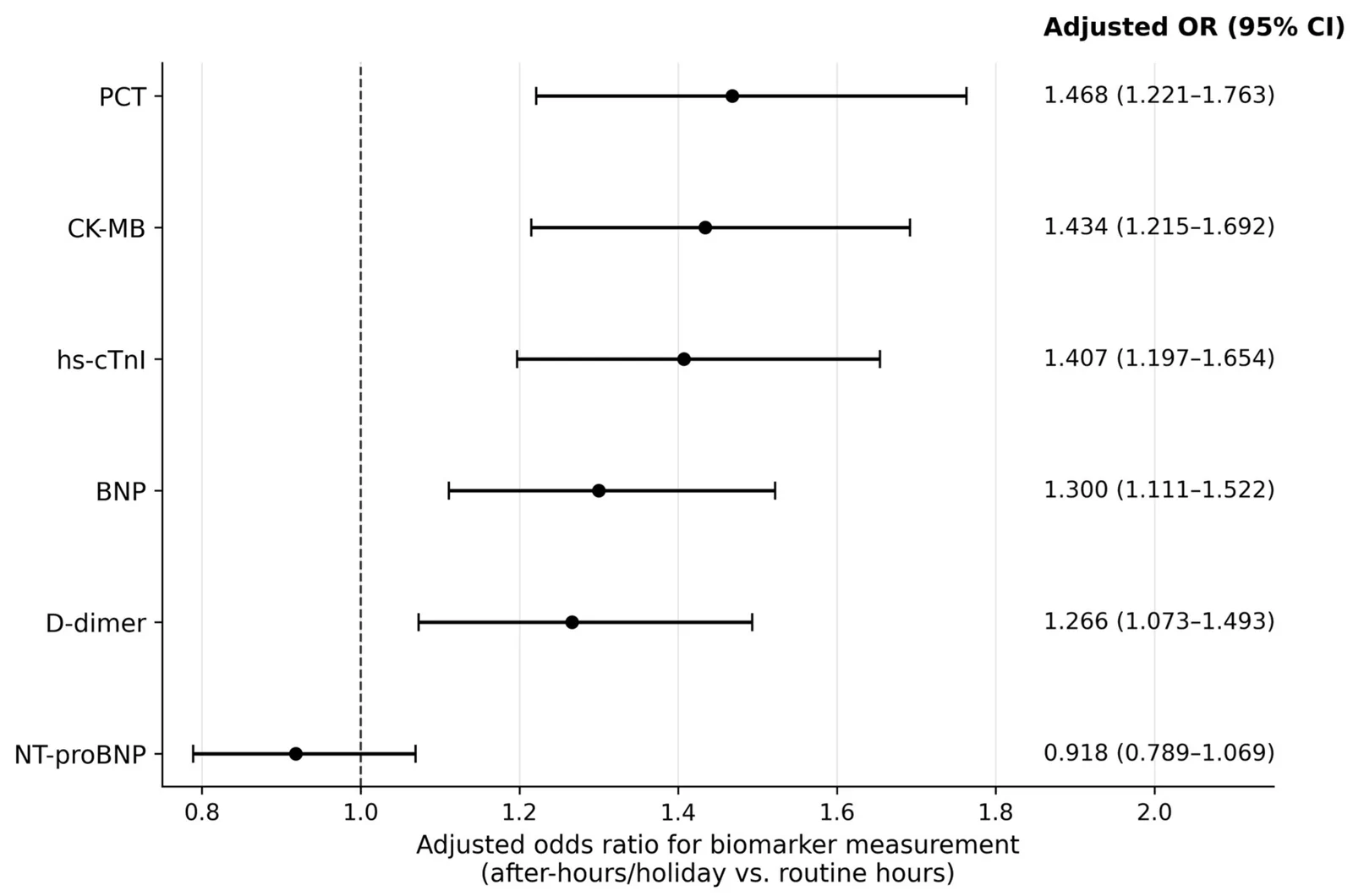
Association between after-hours/holiday context and biomarker measurement. Points indicate adjusted odds ratios and horizontal lines indicate 95% confidence intervals. Models included age, sex, requesting department, calendar month, and calendar year. Routine hours served as the reference category. Abbreviations: PCT, procalcitonin; CK-MB, creatine kinase-MB; hs-cTnI, high-sensitivity cardiac troponin I; BNP, B-type natriuretic peptide; NT-proBNP, N-terminal pro-B-type natriuretic peptide.

After adjustment for age, sex, department, month, and year, after-hours/holiday episodes had higher odds of PCT measurement (OR, 1.468; 95% CI, 1.221–1.763), CK-MB measurement (1.434; 1.215–1.692), hs-cTnI measurement (1.407; 1.197–1.654), BNP measurement (1.300; 1.111–1.522), and D-dimer measurement (1.266; 1.073–1.493). NT-proBNP measurement was not independently associated with operational time (OR, 0.918; 95% CI, 0.789–1.069). Adjusted associations are shown in Figure 3.

### 3.7. Multivariable Associations with Biomarker Measurement

Pronounced department-specific heterogeneity persisted after adjustment for age, sex, operational time, calendar month, and calendar year. Adjusted associations with department, age, and operational time are presented in Table 6 and visualized in Supplementary Figure S4.

Compared with Pulmonology, Pediatrics was associated with markedly lower odds of PCT (OR, 0.109), CK-MB (0.036), hs-cTnI (0.161), BNP (0.068), D-dimer (0.045), and NT-proBNP (0.340) measurement.

Conversely, Cardiology showed higher odds of CK-MB (OR, 2.090), hs-cTnI (2.030), and NT-proBNP (2.415) measurement. Infectious Diseases showed higher odds of CK-MB, hs-cTnI, BNP, D-dimer, and NT-proBNP measurement. Increasing age was independently associated with higher odds of measurement of all six selectively measured biomarkers.

### 3.8. Observed Clinical and Operational Variables and Added Biomarker Concentration

In the complete cohort, the model containing age, sex, calendar month, requesting department, operational time, and calendar year had an apparent AUC of 0.8263. Biomarker concentrations were evaluated within their respective measured subsets, using the same observations for each base and extended model comparison.

Within biomarker-measured cohorts, apparent base-model AUCs varied from 0.7652 for D-dimer to 0.8254 for CRP. These AUCs were estimated separately in each measured subset and should not be compared as if they arose from the same analytical population.

Adding the corresponding biomarker concentration produced little additional discrimination. The largest ΔAUC was 0.00418 for D-dimer. All biomarkers other than D-dimer showed ΔAUC values < 0.001, indicating negligible incremental discrimination.

**Figure 4:**
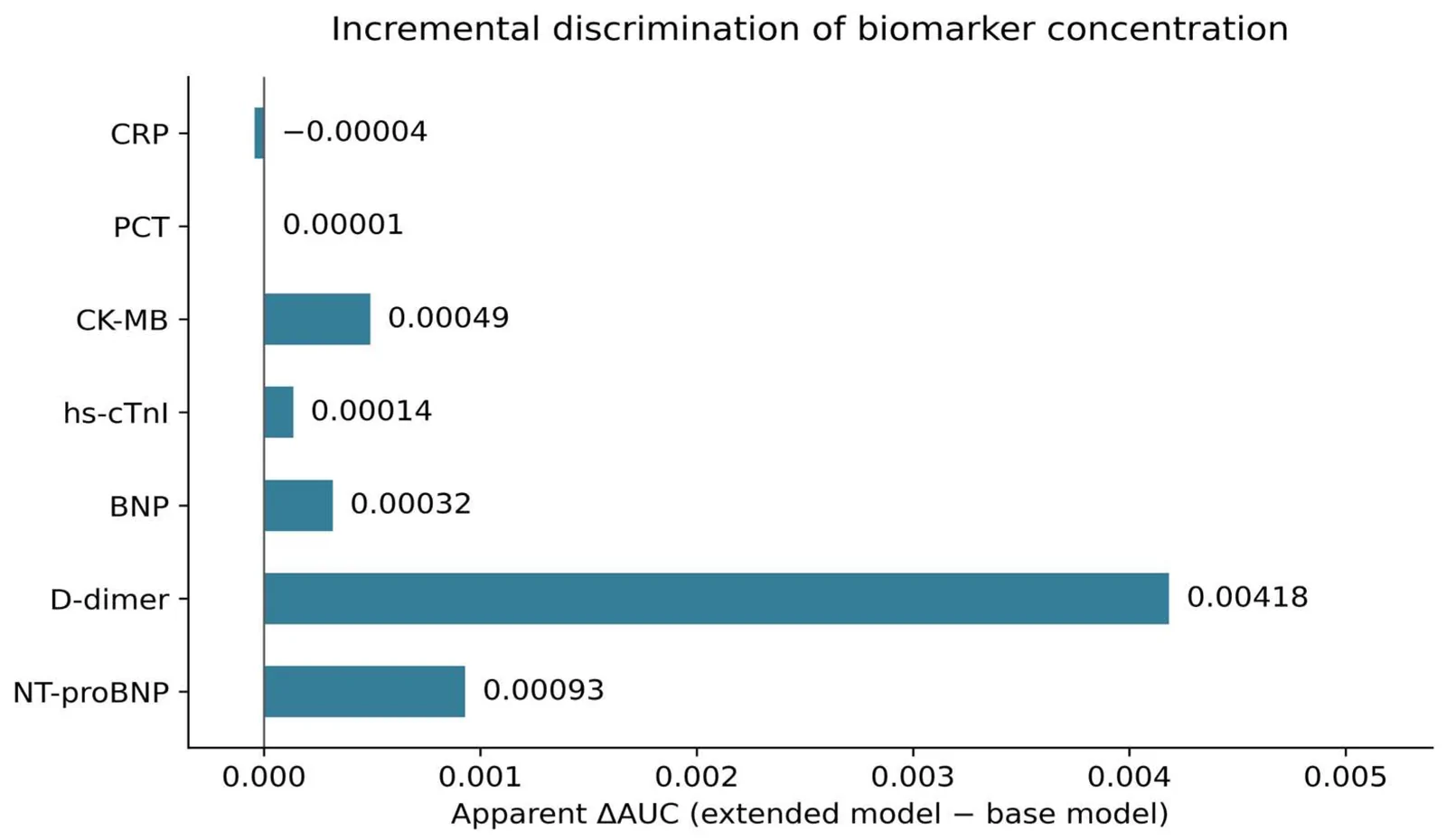
Incremental discrimination after adding biomarker concentration to the base model. Bars show ΔAUC, calculated as the extended-model AUC minus the base-model AUC using unrounded values within the same biomarker-measured cohort. Base models included age, sex, requesting department, operational time, calendar month, and calendar year. Extended models added log2-transformed biomarker concentration. The increments are descriptive apparent estimates and are not tests of clinical utility. Abbreviations: AUC, area under the receiver operating characteristic curve; CRP, C-reactive protein; PCT, procalcitonin; CK-MB, creatine kinase-MB; hs-cTnI, high-sensitivity cardiac troponin I; BNP, B-type natriuretic peptide; NT-proBNP, N-terminal pro-B-type natriuretic peptide.

D-dimer remained independently associated with respiratory viral positivity in the full model; a two-fold increase was associated with an adjusted OR of 0.890 (95% CI, 0.826–0.960; *p* = 0.002). The 95% confidence intervals for the other biomarker concentration estimates included the null value of 1.0. Biomarker-specific base and extended models are summarized in Table 7, and corresponding ΔAUC values are shown in Figure 4.

The extended concentration models contained 27 slope parameters. Positive events per parameter ranged from 6.07 for BNP to 35.74 for CRP, with a value of 7.22 for NT-proBNP, indicating limited information relative to model complexity in some subsets. The repeated cross-validation results are summarized in Supplementary Table S4. Mean out-of-fold ΔAUC was 0.00367 for D-dimer and ranged from −0.00061 to 0.00036 for the other biomarkers. No convergence warnings occurred in the 350 cross-validation fits.

### 3.9. Inverse Probability Weighting

IPW shifted estimated positivity rates after accounting for the observed covariates. Estimated positivity changed from 8.83% to 10.20% for PCT, 8.01% to 11.06% for CK-MB, 8.26% to 11.07% for hs-cTnI, 9.28% to 10.87% for BNP, 8.54% to 8.93% for D-dimer, and 7.99% to 10.77% for NT-proBNP (Supplementary Table S2). These changes are consistent with observed differences between measured and unmeasured episodes but do not establish their causal explanation.

Weighting stability varied substantially. ESS was 1363/4608 (29.6%) for CK-MB, 645/1767 (36.5%) for BNP, and 1663/4787 (34.7%) for D-dimer. Maximum stabilized weights were 35.71, 22.87, and 27.05, respectively. Capping weights at the 1st and 99th percentiles changed positivity from 11.06% to 8.61%, 10.87% to 8.54%, and 8.93% to 8.28%, respectively. For BNP, 626 episodes had an estimated measurement probability below 0.01, including only three measured episodes. Weight-distribution and overlap diagnostics are provided in Supplementary Table S6 and Figures S2 and S3. CRP-weighted estimates are not reported because of separation concerns in its measurement-probability model.

### 3.10. Sensitivity Analyses

Flexible age modeling did not materially alter the principal concentration findings (Supplementary Table S3). In the complete cohort, the age nonlinearity likelihood-ratio test yielded χ^2^ = 9.506 with 2 degrees of freedom (*p* = 0.0086), and the base-model AUC changed from 0.8263 to 0.8274. With spline-adjusted age, the D-dimer OR was 0.890 (95% CI, 0.826–0.960; *p* = 0.003), with a ΔAUC of 0.00429. The corresponding PCT measurement-status OR was 0.687 (95% CI, 0.570–0.828; *p* < 0.001); the D-dimer measurement-status association was weaker (OR, 0.829; 95% CI, 0.679–1.012; *p* = 0.066). The corresponding measurement-status estimates are presented in Supplementary Table S8. Categorical-age concentration models showed numerical instability in some subsets and were not treated as confirmatory sensitivity evidence.

Explicit lower- or upper-limit flags are summarized in Supplementary Table S5. Of 4787 D-dimer measurements, 213 (4.45%) were above the reported upper limit. Excluding these yielded *n* = 4574, an OR of 0.883 (95% CI, 0.813–0.959; *p* = 0.003), and ΔAUC 0.00402. Exclusion of exact-record duplicate candidates and the sine/cosine seasonality specification produced similar D-dimer concentration estimates (Supplementary Table S7).

Likelihood-ratio tests comparing linear and spline terms for log2 biomarker concentrations, with spline-adjusted age, yielded *p* values from 0.067 to 0.785. These exploratory tests did not provide strong evidence of concentration nonlinearity but did not establish linearity.

## 4. Discussion

### 4.1. Principal Findings

Biomarker availability in this cohort was associated with the observed demographic, departmental, and temporal variables. After adjustment for these variables, biomarker concentrations added little discrimination for respiratory viral positivity. The largest apparent ΔAUC was 0.00418 for D-dimer, and the corresponding mean out-of-fold increment was 0.00367.

The principal implication is methodological. Biomarker availability and concentration address different analytical questions, and analyses restricted to measured episodes may describe selected subgroups. The observed patterns are compatible with healthcare-process bias and informative missingness [18,19,20], but the data do not identify the clinical indication for each test or establish the complete selection mechanism.

### 4.2. Age and Calendar Period as Contextual Covariates

Age was retained because respiratory-virus epidemiology varies across age groups [25,26,27,28]. Previous systematic reviews have examined viral co-detection in relation to disease severity [29,30], an outcome that was not assessed in the present study. Calendar month and year were retained to account for temporal variation, including seasonality and pandemic-related disruptions [1,2,31,32,33,34]. These epidemiologic patterns, including age-, sex-, and co-detection-related findings, were addressed previously in the overlapping CRP-available archive [21] and are not reinterpreted here as novel findings. Reported sex-related differences in antiviral immunity [35,36] further support treating sex as a contextual adjustment variable rather than assigning a causal interpretation to the modest crude difference observed in this cohort.

The additional spline analysis suggested that the relationship between age and viral positivity was not fully linear in the complete cohort. Nevertheless, the concentration findings were similar after flexible age adjustment. Sparse age–department combinations made some categorical-age models unstable, supporting emphasis on the spline-based sensitivity results rather than treating numerical convergence alone as evidence of model stability.

### 4.3. Clinical Rationale for Cardiac and Thrombotic Biomarkers

Cardiac and thrombotic biomarkers were included because acute respiratory infection can precipitate cardiovascular stress, myocardial injury, and heart failure and prompt evaluation for thromboembolism [11,12,13,14,15,16,17,37,38,39,40]. Their measurement in this cohort therefore reflects clinically plausible concerns, but these tests were not expected to function as direct classifiers of respiratory viral infection.

Their concentrations are also strongly context-dependent: cardiac troponin and natriuretic peptide distributions vary with age, sex, cardiovascular disease, and other comorbid conditions [16,41,42]. Accordingly, the limited incremental discrimination for viral positivity observed here does not diminish the value of these biomarkers for their intended cardiovascular indications.

### 4.4. Department as a Marker of Clinical Testing Context

Department-level differences provide context for the biomarker measurement patterns observed in this cohort. Pediatrics had the highest proportion of respiratory-virus-positive episodes but the lowest rates of cardiac and thrombotic testing. Conversely, Cardiology had very high measurement rates of hs-cTnI, CK-MB, and NT-proBNP but low respiratory-virus positivity.

A biomarker-measured cohort should therefore not be assumed to represent the full RT-PCR cohort with randomly missing values removed. It differs on the observed variables and may also differ in unrecorded clinical indications and illness severity.

This phenomenon is consistent with informed presence in electronic health record research. The number and type of interactions with a healthcare system can influence the probability that a variable is observed [43], and the clinical observation process itself may therefore affect inference [44]. Similar concerns have been demonstrated specifically for missing laboratory data [45,46].

Reliable use of routinely collected clinical information as real-world evidence requires attention to data provenance and to how measurements enter the analytical dataset [47]. The department-level differences observed here are therefore part of the data-generating process and should be modeled rather than treated as incidental variation.

### 4.5. Operational Time and Test Utilization

Operational time showed a different pattern from requesting department. Viral positivity was almost identical during routine hours, after-hours, and holidays, but several biomarkers were more likely to be measured during after-hours/holiday testing. Most episodes in the combined after-hours/holiday category occurred on Sundays or public holidays. The pooled estimate therefore does not isolate an association with weekday evening or night-time testing.

After adjustment for age, sex, department, month, and year, PCT, CK-MB, hs-cTnI, BNP, and D-dimer were more frequently measured in episodes accessioned outside routine hours. Operational time was associated with ancillary-test availability but did not establish the timing or indication of individual biomarker orders.

The available timestamp represented RT-PCR laboratory accession rather than the ordering, collection, or reporting time of each biomarker. It defined episode-level operational time but could not establish whether a biomarker was ordered or resulted before or after RT-PCR sampling or reporting. The analysis therefore supports an association between operational timing and biomarker availability, without establishing the chronology or causes of individual testing decisions.

### 4.6. CRP and PCT as Contextual Inflammatory Biomarkers

CRP is a broad inflammatory marker rather than an etiologic classifier [9], and PCT, although more closely linked to bacterial infection, can be increased in nonbacterial and noninfectious conditions. Systematic evaluations likewise show limited etiologic discrimination when CRP or PCT is used in isolation in heterogeneous emergency or respiratory populations [48,49,50,51].

Antibiotic stewardship addresses a different clinical question from discrimination of respiratory viral RT-PCR positivity. In a retrospective study of patients with influenza or RSV, PCT testing was not associated with a statistically significant reduction in antibiotic use or dosage [52]. Expert recommendations discuss the implementation of CRP point-of-care testing to support antibiotic prescribing in primary care [53,54]. A French randomized trial did not find a statistically significant reduction in overall antibiotic prescribing [55], whereas a Vietnamese cluster-randomized trial reported lower prescribing with a CRP-testing intervention [56]. CRP testing has also been discussed alongside complementary stewardship strategies in children [57]. The present study did not evaluate antibiotic prescribing or treatment outcomes.

PCT result availability and PCT concentration represented different exposures evaluated in different analytical populations. Result availability was evaluated in the complete cohort, whereas concentration was evaluated only among episodes with a PCT result. The measurement-status OR changed from 0.571 after adjustment for age, sex, and month to 0.702 after adding department, operational time, and year, indicating attenuation after adjustment for additional observed variables. In contrast, the fully adjusted OR per doubling of PCT concentration was 0.991 (95% CI, 0.953–1.030; *p* = 0.631). These ORs should not be compared as estimates of the same effect. The measurement-status association should not be attributed to an intrinsic effect of PCT concentration or interpreted as evidence that the complete clinical ordering process was captured.

### 4.7. D-dimer and the Heterogeneous RT-PCR-Negative Group

D-dimer was the only concentration that remained consistently associated with respiratory viral positivity after progressively more extensive adjustment, but the direction was inverse. This should not be interpreted as evidence that respiratory viral infection suppresses coagulation activation.

D-dimer concentrations increase with age and in numerous clinical states, including inflammation, malignancy, hospitalization, and suspected thromboembolism; interpretation therefore depends on pretest probability [17,58,59]. The RT-PCR-negative group was clinically heterogeneous and may have included bacterial infection, malignancy, decompensated heart failure, suspected pulmonary embolism, postoperative states, or other severe nonviral illnesses.

The inverse association may reflect case mix or unmeasured indications for testing, but the available data cannot distinguish these explanations. Its apparent ΔAUC decreased from 0.01108 in the age-, sex-, and month-adjusted model to 0.00418 in the full model, and the mean out-of-fold increment was 0.00367. These small increments do not support interpreting D-dimer as a biomarker for viral detection.

### 4.8. Statistical Association Is Not Equivalent to Diagnostic Discrimination

A statistically detectable association does not necessarily imply a useful improvement in discrimination. The biomarker-specific apparent base-model AUCs and their small changes after adding concentrations reflect different aspects of model performance than the concentration ORs [60,61,62,63]. Interpretation therefore considered effect estimates, uncertainty, and incremental discrimination together.

ΔAUC remained a descriptive measure; no formal correlated-AUC significance test was performed [64]. Repeated cross-validation provided an internal check on apparent discrimination and showed only small incremental contributions. These analyses do not establish clinical predictive utility: patient-level separation could not be ensured, and neither temporal nor external validation was performed.

### 4.9. Selection Bias, Missing Data, and IPW

Conditioning an analysis on whether a laboratory test was measured can generate selection bias when the same clinical factors influence both test availability and the outcome [65]. Here, biomarker-measured and biomarker-unmeasured groups differed substantially in age, department distribution, and viral positivity.

IPW moved several positivity estimates toward the complete-cohort value, consistent with differences on the observed covariates. However, CK-MB, BNP, and D-dimer had ESS values of only 29.6%, 36.5%, and 34.7% of their measured sample sizes, respectively, and weight capping materially changed their estimates. The BNP overlap diagnostic additionally identified a low-probability region with few measured episodes. These results limit the stability and scope of weighted comparisons.

IPW should therefore be regarded as a descriptive sensitivity analysis conditional on the observed variables, not as a means of recovering unmeasured concentrations or removing unmeasured confounding [24]. Missing clinical indications, severity, renal function, and other determinants may affect both testing and positivity. Multiple imputation would not by itself resolve missingness generated by incompletely measured clinical processes [66,67].

### 4.10. Diagnostic Stewardship Implications

These results have methodological implications for retrospective laboratory research and diagnostic stewardship [20,68]. Utilization patterns and measured concentrations provide complementary descriptions of the available laboratory data; their associations with viral positivity should be interpreted within the study’s methodological scope.

Studies using routinely collected laboratory data should describe how results become available, specify the covariates used to assess selective measurement, and acknowledge uncertainty regarding missing clinical indications and test timing. The current findings do not determine whether an individual biomarker should be ordered for a particular patient.

### 4.11. Strengths and Limitations

Strengths include the use of a large real-world cohort; simultaneous evaluation of seven inflammatory, cardiac, and thrombotic biomarkers; explicit modeling of biomarker availability; and complementary analyses of department, operational time, incremental discrimination, IPW, and sensitivity to age specification and D-dimer censoring.

Treating biomarker availability as part of the data-generating process is another strength. This approach links clinical-biochemistry data analysis with informed presence and diagnostic stewardship concepts [18,19,20,43,44,45,46,47,68].

Several limitations require emphasis. First, this retrospective, single-center study evaluated observed associations and cannot establish causal effects or the clinical reasons for ordering individual tests. The RT-PCR-negative group was clinically heterogeneous, and positivity was used as a recorded reference outcome rather than an intended diagnostic target of the biomarkers.

Second, the analytical unit was a released RT-PCR record. Patient, encounter, and specimen identifiers were unavailable, so the number of unique patients, within-patient dependence, and true duplicate specimens could not be determined. Exact-record exclusion does not resolve these issues. Conventional confidence intervals and *p* values may overstate precision when episodes are correlated, and patient-level separation could not be ensured during cross-validation.

Third, linkage used a ±24-h reporting-time criterion. Individual biomarker reporting, ordering, and collection timestamps were unavailable, preventing independent linkage verification, narrower-window analyses, and assessment of whether measurements preceded or followed RT-PCR testing. The episode-level accession timestamp describes operational timing but does not establish the chronology of clinical decisions or biological changes.

Fourth, cardiovascular history, renal function, bacterial coinfection, suspected pulmonary embolism, medications, intensive care status, illness severity, and outcomes were unavailable. The models therefore represent selected observed clinical and operational variables rather than the complete decision-making process. Only 12 episodes carried an Emergency Medicine requesting code, which may not capture all emergency care; a reliable adjusted emergency-department subgroup analysis was not feasible.

Fifth, hMPV and enterovirus were unavailable under the approved dataset, and their unperformed or missing-result counts could not be distinguished. SARS-CoV-2 was not included. Positivity therefore represented only the available 13-target panel, and RT-PCR detection does not necessarily establish symptomatic causation [69]. Positivity was based on the laboratory’s assay-specific Ct < 27 criterion. Raw Ct values and longitudinal assay, reagent, and protocol-change records were unavailable for independent assessment of analytical comparability across targets and time.

Finally, some biomarker subsets had few events relative to model complexity, particularly BNP and NT-proBNP. Some categorical-age concentration models were numerically unstable. The reported spline analyses addressed the complete-cohort viral-positivity model, biomarker concentration models, and selected measurement-status associations with viral positivity; they should not be interpreted as validation of every model of biomarker availability. Internal cross-validation was exploratory and does not substitute for temporal or external validation [62,63]. The findings should not be interpreted as evidence for or against ordering these biomarkers for their intended indications in individual patients.

## 5. Conclusions

In this tertiary-care RT-PCR cohort, biomarker availability was associated with observed demographic, departmental, and operational variables. PCT measurement status and PCT concentration showed different patterns, underscoring the need to distinguish test availability from the value obtained when the test is performed.

Biomarker concentrations provided little additional discrimination for viral positivity within the available respiratory viral panel. D-dimer retained an inverse association, but its apparent and cross-validated incremental discrimination was small. These findings do not imply that the biomarkers lack value for their intended inflammatory, cardiovascular, or thrombotic indications.

Retrospective laboratory studies should report how measurements become available, compare measured and unmeasured episodes, and assess the stability of analyses that depend on selective testing. The present findings are methodological and are limited by unmeasured clinical indications and the inability to identify repeated patients or reconstruct individual test timing.

## Figures and Tables

**Table 1:** Baseline demographic, virological, and biomarker characteristics.

Characteristic	Value
Total testing episodes	7726
Age, median (IQR), years	72 (58–80)
Male	4643 (60.10%)
Female	3083 (39.90%)
Viral positive	983 (12.72%)
Viral negative	6743 (87.28%)
Single viral detection	854 (11.05%)
Dual viral detections	112 (1.45%)
≥3 viral detections	17 (0.22%)
CRP, mg/dL	8.95 (2.78–15.41); *n* = 7639
PCT, ng/mL	0.295 (0.118–1.120); *n* = 5858
CK-MB, ng/mL	2.20 (1.10–4.30); *n* = 4608
hs-cTnI, pg/mL	22.60 (9.10–77.58); *n* = 4672
BNP, pg/mL	158 (74–404); *n* = 1767
D-dimer, µg/mL FEU	2.36 (1.24–4.41); *n* = 4787
NT-proBNP, pg/mL	1696 (435.65–6537); *n* = 2441

Abbreviations: IQR, interquartile range; CRP, C-reactive protein; PCT, procalcitonin; CK-MB, creatine kinase-MB; hs-cTnI, high-sensitivity cardiac troponin I; BNP, B-type natriuretic peptide; NT-proBNP, N-terminal pro-B-type natriuretic peptide; FEU, fibrinogen-equivalent units.

**Table 2:** Biomarker availability and composition of measured and unmeasured cohorts.

Biomarker	Measured, *n* (%)	Median Age: Measured vs. Unmeasured	Positivity: Measured	Positivity: Unmeasured
CRP	7639 (98.87)	72 vs. 59	12.63%	20.69%
PCT	5858 (75.82)	74 vs. 62	8.83%	24.95%
CK-MB	4608 (59.64)	76 vs. 63	8.01%	19.69%
hs-cTnI	4672 (60.47)	75 vs. 64	8.26%	19.55%
BNP	1767 (22.87)	76 vs. 70	9.28%	13.74%
D-dimer	4787 (61.96)	75 vs. 64	8.54%	19.53%
NT-proBNP	2441 (31.59)	75 vs. 70	7.99%	14.91%

Abbreviations: CRP, C-reactive protein; PCT, procalcitonin; CK-MB, creatine kinase-MB; hs-cTnI, high-sensitivity cardiac troponin I; BNP, B-type natriuretic peptide; NT-proBNP, N-terminal pro-B-type natriuretic peptide. Note: Measured and unmeasured groups were defined separately for each biomarker. The measured percentage uses all 7726 episodes as the denominator. Each positivity percentage uses the number of episodes in that biomarker-specific measured or unmeasured group as its denominator and represents detection of at least one of the 13 included respiratory viral targets.

**Table 3:** CRP and PCT availability, concentration summaries, and discrimination.

Analysis	CRP	PCT
Available testing episodes	7639	5858
Median (IQR)	8.95 (2.78–15.41) mg/dL	0.295 (0.118–1.120) ng/mL
Univariate AUC (joint complete cases, *n* = 5849)	0.5029	0.4898
Base-model AUC (joint complete cases, *n* = 5849)	0.6758	0.6758
Biomarker-added AUC	0.6787	0.6760

Abbreviations: CRP, C-reactive protein; PCT, procalcitonin; IQR, interquartile range; AUC, area under the receiver operating characteristic curve; Note: Availability counts and concentration summaries refer to each biomarker-specific measured cohort. All AUCs in this table were calculated in the same 5849 episodes with both CRP and PCT measurements. The base model included age, sex, and categorical calendar month. CRP and PCT were entered as untransformed concentrations. The model containing both had an AUC of 0.6797. These specifications differ from the subsequent log2-transformed concentration models.

**Table 4:** Cardiac and thrombotic biomarker associations and discrimination under age-, sex-, and month-adjusted modeling.

Biomarker	*n*	Univariate AUC	Adjusted OR Per 2-Fold Increase	*p* Value	Base AUC	Extended AUC	ΔAUC
CK-MB	4608	0.5057	1.031 (0.963–1.103)	0.387	0.6629	0.6636	0.00073
hs-cTnI	4672	0.4897	1.015 (0.975–1.057)	0.472	0.6716	0.6715	−0.00009
BNP	1767	0.4777	0.939 (0.855–1.031)	0.187	0.6846	0.6888	0.00413
D-dimer	4787	0.4423	0.872 (0.812–0.938)	<0.001	0.6632	0.6743	0.01108
NT-proBNP	2441	0.5128	1.050 (0.993–1.110)	0.088	0.6749	0.6775	0.00259

Abbreviations: AUC, area under the receiver operating characteristic curve; OR, odds ratio; CK-MB, creatine kinase-MB; hs-cTnI, high-sensitivity cardiac troponin I; BNP, B-type natriuretic peptide; NT-proBNP, N-terminal pro-B-type natriuretic peptide. Note: Adjusted ORs represent the change in positivity odds per two-fold increase in concentration. Base models included age, sex, and categorical calendar month and were fitted to the same measured episodes as the corresponding biomarker-added models. AUCs are displayed to four decimal places, and ΔAUC to five; ΔAUC was calculated from unrounded AUCs.

**Table 5:** Biomarker measurement rates by requesting department.

Department	*n*	Age, Median	Positivity (%)	CRP	PCT	CK-MB	hs-cTnI	BNP	D-dimer	NT-proBNP
Pulmonology	2863	76	8.6%	98.8%	82.6%	76.2%	76.4%	32.5%	80.0%	38.5%
Hematology	1428	68	10.3%	99.6%	88.3%	38.9%	39.0%	13.0%	61.3%	16.1%
Pediatrics	837	1	49.2%	96.9%	23.5%	2.3%	9.6%	0.8%	4.4%	9.2%
Nephrology	700	77	7.9%	100.0%	86.7%	77.9%	77.7%	28.3%	65.3%	34.6%
Cardiology	458	79	6.6%	99.3%	84.1%	87.1%	86.9%	16.4%	67.2%	60.5%
Infectious Diseases	418	68	2.2%	100.0%	75.4%	83.0%	83.0%	38.8%	82.8%	52.4%
Other Internal Medicine	531	72	9.0%	98.3%	80.4%	54.0%	54.0%	16.4%	50.5%	30.3%
Other Departments	491	76	7.5%	98.2%	61.1%	55.6%	55.6%	25.1%	42.0%	27.1%

Abbreviations: CRP, C-reactive protein; PCT, procalcitonin; CK-MB, creatine kinase-MB; hs-cTnI, high-sensitivity cardiac troponin I; BNP, B-type natriuretic peptide; NT-proBNP, N-terminal pro-B-type natriuretic peptide. Note: Values for biomarker measurement are percentages of RT-PCR testing episodes within each requesting-department category. Other Internal Medicine comprised Gastroenterology, Rheumatology, and Endocrinology. All remaining departments were classified as Other Departments as defined in the Methods.

**Table 6:** Adjusted associations with biomarker measurement.

Predictor	PCT, OR (95% CI); *p*	CK-MB, OR (95% CI); *p*	hs-cTnI, OR (95% CI); *p*	BNP, OR (95% CI); *p*	D-dimer, OR (95% CI); *p*	NT-proBNP, OR (95% CI); *p*
Age, per 10 years	1.105 (1.057–1.155); <0.001	1.255 (1.207–1.305); <0.001	1.254 (1.206–1.304); <0.001	1.175 (1.124–1.229); <0.001	1.175 (1.130–1.222); <0.001	1.119 (1.076–1.165); <0.001
After-hours/holiday	1.468 (1.221–1.763); <0.001	1.434 (1.215–1.692); <0.001	1.407 (1.197–1.654); <0.001	1.300 (1.111–1.522); 0.001	1.266 (1.073–1.493); 0.005	0.918 (0.789–1.069); 0.269
Hematology	1.648 (1.356–2.003); <0.001	0.234 (0.203–0.269); <0.001	0.233 (0.202–0.268); <0.001	0.371 (0.310–0.444); <0.001	0.467 (0.403–0.541); <0.001	0.336 (0.285–0.395); <0.001
Pediatrics	0.109 (0.076–0.158); <0.001	0.036 (0.021–0.061); <0.001	0.161 (0.111–0.232); <0.001	0.068 (0.030–0.155); <0.001	0.045 (0.029–0.069); <0.001	0.340 (0.233–0.495); <0.001
Nephrology	1.411 (1.108–1.798); 0.005	1.105 (0.904–1.351); 0.330	1.089 (0.891–1.332); 0.406	0.828 (0.686–0.998); 0.048	0.464 (0.385–0.559); <0.001	0.827 (0.694–0.985); 0.033
Cardiology	1.023 (0.779–1.343); 0.870	2.090 (1.564–2.793); <0.001	2.030 (1.522–2.708); <0.001	0.438 (0.336–0.571); <0.001	0.545 (0.436–0.680); <0.001	2.415 (1.968–2.964); <0.001
Infectious Diseases	0.626 (0.484–0.808); <0.001	1.900 (1.434–2.518); <0.001	1.873 (1.414–2.481); <0.001	2.076 (1.650–2.612); <0.001	1.832 (1.383–2.426); <0.001	1.839 (1.480–2.284); <0.001
Other Internal Medicine	0.870 (0.684–1.107); 0.257	0.419 (0.344–0.510); <0.001	0.415 (0.341–0.504); <0.001	0.473 (0.369–0.606); <0.001	0.288 (0.236–0.351); <0.001	0.740 (0.604–0.906); 0.004
Other Departments	0.316 (0.256–0.391); <0.001	0.413 (0.338–0.506); <0.001	0.412 (0.336–0.504); <0.001	0.753 (0.601–0.943); 0.014	0.184 (0.149–0.226); <0.001	0.592 (0.477–0.734); <0.001

Abbreviations: OR, odds ratio; CI, confidence interval; PCT, procalcitonin; CK-MB, creatine kinase-MB; hs-cTnI, high-sensitivity cardiac troponin I; BNP, B-type natriuretic peptide; NT-proBNP, N-terminal pro-B-type natriuretic peptide. Note: Separate multivariable logistic regression models were constructed for each biomarker, with biomarker measurement status as the dependent variable. All models included age, sex, requesting department, operational time, calendar month, and calendar year. Pulmonology was the reference requesting department, and routine hours were the reference operational-time category.

**Table 7:** Incremental discrimination beyond the observed clinical and operational variables.

Biomarker	*n* (Positive/Negative)	Adjusted OR Per 2-Fold Increase (95% CI)	*p* Value	Base AUC	Extended AUC	ΔAUC
CRP	7639 (965/6674)	0.988 (0.946–1.032)	0.589	0.8254	0.8254	−0.00004
PCT	5858 (517/5341)	0.991 (0.953–1.030)	0.631	0.7784	0.7784	0.00001
CK-MB	4608 (369/4239)	1.050 (0.976–1.129)	0.192	0.7772	0.7777	0.00049
hs-cTnI	4672 (386/4286)	1.024 (0.978–1.072)	0.314	0.7820	0.7821	0.00014
BNP	1767 (164/1603)	0.963 (0.870–1.065)	0.461	0.7843	0.7847	0.00032
D-dimer	4787 (409/4378)	0.890 (0.826–0.960)	0.002	0.7652	0.7694	0.00418
NT-proBNP	2441 (195/2246)	1.040 (0.973–1.111)	0.246	0.7918	0.7927	0.00093

Abbreviations: OR, odds ratio; CI, confidence interval; AUC, area under the receiver operating characteristic curve; CRP, C-reactive protein; PCT, procalcitonin; CK-MB, creatine kinase-MB; hs-cTnI, high-sensitivity cardiac troponin I; BNP, B-type natriuretic peptide; NT-proBNP, N-terminal pro-B-type natriuretic peptide. Note: The base model included age, sex, requesting department, operational time, calendar month, and calendar year. The extended model added the corresponding log2-transformed biomarker concentration in the identical measured subset. ORs represent associations per two-fold increase in concentration. ΔAUC was calculated using unrounded AUC values; the difference between the rounded AUCs displayed in the table may therefore differ from the displayed ΔAUC. AUCs and ΔAUC are descriptive apparent estimates.

## Data Availability

The datasets analyzed in this study are not publicly available because they contain routinely collected clinical laboratory and electronic medical record data subject to institutional and privacy restrictions. De-identified data may be available from the corresponding author upon reasonable request, subject to Institutional Review Board approval and applicable data-use requirements.

## Supplementary Materials

The following supporting information can be downloaded at: https://www.mdpi.com/article/10.3390/diagnostics16193232/s1, Table S1: Biomarker utilization according to operational time; Table S2: Descriptive IPW assessment of biomarker-specific cohorts; Table S3: Sensitivity analysis using restricted cubic splines for age; Table S4: Model specifications, events per parameter, and repeated cross-validation—Panel A: Cohorts and model complexity, —Panel B: Apparent discrimination, —Panel C: Repeated cross-validation; Table S5: Biomarker availability and explicit lower- and upper-limit flags; Table S6: Weight-distribution and overlap diagnostics—Panel A: Stabilized weight quantiles, —Panel B: Effective information and low-probability regions, —Panel C: Unclipped measurement-probability distributions; Table S7: Additional sensitivity and nonlinearity analyses—Panel A: D-dimer sensitivity analyses, —Panel B: Biomarker concentration nonlinearity, —Panel C: Age nonlinearity, —Panel D: Alternative seasonal specification, —Panel E: Concentration spline knots; Table S8: Associations between biomarker result availability and respiratory viral RT-PCR positivity; Figure S1: Distribution of log2-transformed D-dimer concentration according to respiratory viral detection group; Figure S2: Estimated probability of biomarker measurement among measured and unmeasured episodes; Figure S3: Stabilized inverse probability weight distributions among biomarker-measured episodes before percentile capping; Figure S4: Adjusted associations with biomarker measurement; File S1: Estimated measurement-probability distributions (propensity_overlap.csv).

## Abbreviations

The following abbreviations are used in this manuscript:

AUC	Area under the receiver operating characteristic curve
BNP	B-type natriuretic peptide
CI	Confidence interval
CK-MB	Creatine kinase-MB
COVID-19	Coronavirus disease 2019
CRP	C-reactive protein
FEU	Fibrinogen-equivalent units
hs-cTnI	High-sensitivity cardiac troponin I
IPW	Inverse probability weighting
IQR	Interquartile range
NT-proBNP	N-terminal pro-B-type natriuretic peptide
OR	Odds ratio
PCT	Procalcitonin
RSV	Respiratory syncytial virus
RT-PCR	Reverse transcription polymerase chain reaction
