# Supplementary material for: Observed Clinical and Operational Factors Are Associated with Biomarker Test Utilization in Respiratory Virus RT-PCR Testing: A Real-World Analysis of Inflammatory, Cardiac, and Thrombotic Biomarkers
Source: Diagnostics (Basel). 2026 Oct 7;16(19):3232. doi: 10.3390/diagnostics16193232 (PMC13664205; doi:10.3390/diagnostics16193232)
Supplement: Supplementary file 1 [file diagnostics-16-03232-s001.zip › Revised_Supplementary_Materials.pdf]

Supplementary Materials

Observed Clinical and Operational Factors Are Associated with Biomarker Test Utilization in Respiratory Virus RT-PCR Testing: A Real-World Analysis of Inflammatory, Cardiac, and Thrombotic Biomarkers

Yonghee Lee, Ga-Yeon Kim, and Jae Kyung Kim

Supplementary Table S1. Biomarker utilization according to operational time

Operational time	n (%)	Median age, years	Viral positivity, n (%)	CRP measured, n (%)	PCT measured, n (%)	CK-MB measured, n (%)	hs-cTnI measured, n (%)	BNP measured, n (%)	D-dimer measured, n (%)	NT-proBNP measured, n (%)
Routine hours	6,685 (86.53)	72	844 (12.63)	6,605 (98.80)	5,024 (75.15)	3,922 (58.67)	3,979 (59.52)	1,464 (21.90)	4,083 (61.08)	2,133 (31.91)
After-hours	150 (1.94)	73	19 (12.67)	148 (98.67)	108 (72.00)	99 (66.00)	99 (66.00)	50 (33.33)	98 (65.33)	54 (36.00)
Holidays	891 (11.53)	73	120 (13.47)	886 (99.44)	726 (81.48)	587 (65.88)	594 (66.67)	253 (28.40)	606 (68.01)	254 (28.51)
Total	7,726 (100.00)	72	983 (12.72)	7,639 (98.87)	5,858 (75.82)	4,608 (59.64)	4,672 (60.47)	1,767 (22.87)	4,787 (61.96)	2,441 (31.59)

Abbreviations: CRP, C-reactive protein; PCT, procalcitonin; CK-MB, creatine kinase-MB; hs-cTnI, high-sensitivity cardiac troponin I; BNP, B-type natriuretic peptide; NT-proBNP, N-terminal pro-B-type natriuretic peptide. RT-PCR, reverse transcription polymerase chain reaction; n, number of episodes.

Note: Routine hours were defined as 08:00 to <17:00 Monday through Friday and 08:00 to <12:00 on Saturday. After-hours comprised all remaining periods on non-holiday dates. Sundays, official national public holidays, and substitute holidays in the Republic of Korea were classified as holidays. Operational time was classified using the RT-PCR accession timestamp, not from individual biomarker ordering or collection times. Percentages in the n (%) column use all 7,726 episodes; viral-positivity and biomarker-measurement percentages use the episode count in the corresponding row.

Supplementary Table S2. Descriptive IPW assessment of biomarker-specific cohorts

Biomarker	Measured episodes, n	Unweighted viral positivity (%)	IPW-weighted viral positivity (%)	1st–99th percentile-truncated IPW positivity (%)	Effective sample size	Truncated effective sample size
PCT	5,858	8.83	10.20	10.18	4,838	4,874
CK-MB	4,608	8.01	11.06	8.61	1,363	4,112
hs-cTnI	4,672	8.26	11.07	10.66	2,917	3,185
BNP	1,767	9.28	10.87	8.54	645	1,301
D-dimer	4,787	8.54	8.93	8.28	1,663	4,377
NT-proBNP	2,441	7.99	10.77	10.37	1,747	1,810

Abbreviations: IPW, inverse probability weighting; CRP, C-reactive protein; PCT, procalcitonin; CK-MB, creatine kinase-MB; hs-cTnI, high-sensitivity cardiac troponin I; BNP, B-type natriuretic peptide; NT-proBNP, N-terminal pro-B-type natriuretic peptide. ESS, effective sample size; n, number of measured episodes; w_i, stabilized inverse probability weight for episode i; y_i, binary viral-positivity indicator for episode i; Σ, summation over measured episodes.

Note: The probability of biomarker measurement was estimated using age, sex, requesting department, operational time, calendar month, and calendar year. Stabilized inverse probability weights were applied to measured episodes to assess selection of the biomarker-specific analytical cohort. Measurement probabilities were restricted to 0.01–0.99. For truncated estimates, weights were additionally restricted at the 1st and 99th percentiles. The respiratory viral positivity rate in the complete source cohort was 12.72%. These weighted estimates are descriptive sensitivity analyses of observed selection and should not be interpreted as recovery of unmeasured biomarker values or as causal correction of unmeasured confounding. CRP-weighted estimates are not reported because near-universal measurement raised separation concerns in the measurement-probability model. The weighted results do not establish adequate overlap throughout the covariate space. Unweighted positivity uses the biomarker-measured cohort as its denominator. Weighted positivity is $100 \times \Sigma(w_i y_i)/\Sigma w_i$, where $y_i = 1$ for a positive episode and 0 otherwise. Effective sample size is $(\Sigma w_i)^2/\Sigma w_i^2$; truncated estimates use the capped weights. Effective sample size is reported as a rounded count, not a percentage.

Supplementary Table S3. Sensitivity analysis using restricted cubic splines for age

Biomarker	OR (95% CI)	P	Base AUC	Added AUC	ΔAUC
CRP	0.991 (0.948–1.036)	0.686	0.8267	0.8265	−0.00011
PCT	0.990 (0.953–1.029)	0.627	0.7785	0.7786	0.00005
CK-MB	1.050 (0.976–1.129)	0.190	0.7774	0.7780	0.00057
hs-cTnI	1.023 (0.977–1.072)	0.332	0.7823	0.7825	0.00018
BNP	0.966 (0.872–1.069)	0.503	0.7849	0.7854	0.00047
D-dimer	0.890 (0.826–0.960)	0.003	0.7655	0.7698	0.00429
NT-proBNP	1.036 (0.969–1.107)	0.300	0.7922	0.7931	0.00087

Abbreviations: CRP, C-reactive protein; PCT, procalcitonin; CK-MB, creatine kinase-MB; hs-cTnI, high-sensitivity cardiac troponin I; BNP, B-type natriuretic peptide; NT-proBNP, N-terminal pro-B-type natriuretic peptide. OR, odds ratio; CI, confidence interval; AUC, area under the receiver operating characteristic curve; ΔAUC, added-model AUC minus base-model AUC. RCS, restricted cubic spline; log2, base-2 logarithm; P, statistical P value.

Note: Age used four knots at 1, 65, 77, and 89 years (full-cohort 5th, 35th, 65th, and 95th percentiles). Models also included sex, department, operational time, categorical month and year. ORs are per doubling of concentration. Each extended model has 29 slope parameters, excluding the intercept. These are post hoc exploratory analyses. The base model contains the stated covariates; the added model additionally includes log2 biomarker concentration. Both models use the same biomarker-measured episodes. AUCs describe apparent discrimination; ΔAUC is calculated as added-model AUC minus base-model AUC before rounding.

Supplementary Table S4. Model specifications, events per parameter, and repeated cross-validation — Panel A: Cohorts and model complexity

Biomarker	Measured n	Positive events	Slopes*	Events/slopes
CRP	7639	965	27	35.74
PCT	5858	517	27	19.15
CK-MB	4608	369	27	13.67
hs-cTnI	4672	386	27	14.30
BNP	1767	164	27	6.07
D-dimer	4787	409	27	15.15
NT-proBNP	2441	195	27	7.22

Abbreviations: CRP, C-reactive protein; PCT, procalcitonin; CK-MB, creatine kinase-MB; hs-cTnI, high-sensitivity cardiac troponin I; BNP, B-type natriuretic peptide; NT-proBNP, N-terminal pro-B-type natriuretic peptide. n, number of measured episodes; Slopes, number of non-intercept model parameters; Events/slopes, positive events divided by the number of slope parameters; log2, base-2 logarithm.

Note: *Extended-model slopes excluding the intercept: age (1), sex (1), department (7), operational time (1), month (11), year (5), and log2 biomarker (1). The base model omits the biomarker and has 26 slopes. Each model pair is fitted to the same measured observations. Events/slopes = positive episodes / 27; this descriptive ratio does not guarantee stability. Measured n denotes the biomarker-measured cohort; positive events are episodes positive for at least one recorded respiratory viral target.

Supplementary Table S4 — Panel B: Apparent discrimination

Biomarker	Base AUC	Added AUC	ΔAUC
CRP	0.8254	0.8254	−0.00004
PCT	0.7784	0.7784	0.00001
CK-MB	0.7772	0.7777	0.00049
hs-cTnI	0.7820	0.7821	0.00014
BNP	0.7843	0.7847	0.00032
D-dimer	0.7652	0.7694	0.00418
NT-proBNP	0.7918	0.7927	0.00093

Abbreviations: CRP, C-reactive protein; PCT, procalcitonin; CK-MB, creatine kinase-MB; hs-cTnI, high-sensitivity cardiac troponin I; BNP, B-type natriuretic peptide; NT-proBNP, N-terminal pro-B-type natriuretic peptide. AUC, area under the receiver operating characteristic curve; ΔAUC, added-model AUC minus base-model AUC; log2, base-2 logarithm.

Note: Values represent apparent performance in each fitting sample. Base models include age, sex, requesting department, operational time, categorical calendar month and calendar year. Extended models add log2 concentration in the same observations. AUCs are shown to four decimals and ΔAUC to five, calculated before rounding. Negative ΔAUC values indicate a lower apparent AUC in the added model; displayed rounded AUCs need not reproduce the displayed ΔAUC exactly.

Supplementary Table S4 — Panel C: Repeated cross-validation

Biomarker	Mean OOF base AUC	Mean OOF extended AUC	Mean ΔAUC	Range of ΔAUC
CRP	0.8198	0.8194	−0.00037	−0.00060 to −0.00014
PCT	0.7643	0.7640	−0.00026	−0.00068 to -3.62×10^{-7}
CK-MB	0.7562	0.7557	−0.00049	−0.00111 to 0.00001
hs-cTnI	0.7616	0.7610	−0.00061	−0.00131 to 2.42×10^{-6}
BNP	0.7374	0.7370	−0.00038	−0.00133 to 0.00018
D-dimer	0.7476	0.7513	0.00367	0.00327 to 0.00408
NT-proBNP	0.7554	0.7558	0.00036	−0.00094 to 0.00121

Abbreviations: CRP, C-reactive protein; PCT, procalcitonin; CK-MB, creatine kinase-MB; hs-cTnI, high-sensitivity cardiac troponin I; BNP, B-type natriuretic peptide; NT-proBNP, N-terminal pro-B-type natriuretic peptide. AUC, area under the receiver operating characteristic curve; OOF, out-of-fold; ΔAUC, extended-model AUC minus base-model AUC.

Note: Five repetitions of stratified five-fold cross-validation. Identical source records were grouped within folds. The range across repetitions is not a confidence interval. Patient-level separation cannot be guaranteed. OOF predictions are obtained from folds not used to fit the corresponding model. Within each repetition, these predictions are pooled to calculate the AUC. Mean ΔAUC is the mean of the within-repetition extended-minus-base AUC differences; the minimum and maximum of those differences define the reported range.

Supplementary Table S5. Biomarker availability and explicit lower- and upper-limit flags

Biomarker	Measured n	Unmeasured n (%)	< flag n (%)	> flag n (%)
CRP	7639	87 (1.13)	561 (7.34)	0 (0.00)
PCT	5858	1,868 (24.18)	0 (0.00)	0 (0.00)
CK-MB	4608	3,118 (40.36)	0 (0.00)	7 (0.15)
hs-cTnI	4672	3,054 (39.53)	63 (1.35)	34 (0.73)
BNP	1767	5,959 (77.13)	0 (0.00)	18 (1.02)
D-dimer	4787	2,939 (38.04)	0 (0.00)	213 (4.45)
NT-proBNP	2441	5,285 (68.41)	5 (0.20)	86 (3.52)

Abbreviations: CRP, C-reactive protein; PCT, procalcitonin; CK-MB, creatine kinase-MB; hs-cTnI, high-sensitivity cardiac troponin I; BNP, B-type natriuretic peptide; NT-proBNP, N-terminal pro-B-type natriuretic peptide. n, number of episodes; < flag, explicit lower-limit result flag; > flag, explicit upper-limit result flag.

Note: Unmeasured percentages use all 7,726 episodes as the denominator; lower- and upper-limit flag percentages use the biomarker-specific measured count. “Unmeasured” means that no linked result appears in the extracted record; it does not establish whether the test was ordered or performed. Only explicit source flags were counted; zero flags do not prove absence of laboratory-system censoring. Values with < were assigned one-half the reported lower limit, values with > were assigned the reported upper limit, and unflagged numeric values were retained. The < and > symbols denote explicit lower- and upper-limit result flags, respectively; they do not denote statistical significance thresholds.

Supplementary Table S6. Weight-distribution and overlap diagnostics — Panel A: Stabilized weight quantiles

Biomarker	Min	P1	Median	P99	Max
PCT	0.799	0.816	0.893	3.703	4.229
CK-MB	0.627	0.652	0.778	2.328	35.712
hs-cTnI	0.638	0.663	0.789	5.835	9.158
BNP	0.310	0.369	0.721	3.141	22.871
D-dimer	0.648	0.673	0.820	2.416	27.053
NT-proBNP	0.414	0.467	0.814	3.628	5.591

Abbreviations: PCT, procalcitonin; CK-MB, creatine kinase-MB; hs-cTnI, high-sensitivity cardiac troponin I; BNP, B-type natriuretic peptide; NT-proBNP, N-terminal pro-B-type natriuretic peptide. IPW, inverse probability weighting; Min, minimum; Max, maximum; P1, 1st percentile; P99, 99th percentile.

Note: Stabilized weights are summarized among measured episodes; estimated measurement probabilities were clipped to 0.01–0.99 before weight calculation. P1/P99 use linear quantile interpolation. Min and Max denote the minimum and maximum stabilized weights, respectively; P1 and P99 denote the 1st and 99th percentiles. Summaries precede percentile capping.

Supplementary Table S6 — Panel B: Effective information and low-probability regions

Biomarker	ESS/n (%)	Top 1% weight share (%)	p < 0.01: all	p < 0.01: measured
PCT	82.6	3.9	0	0
CK-MB	29.6	11.5	0	0
hs-cTnI	62.4	7.1	0	0
BNP	36.5	10.3	626	3
D-dimer	34.7	12.6	0	0
NT-proBNP	71.6	4.5	0	0

Abbreviations: PCT, procalcitonin; CK-MB, creatine kinase-MB; hs-cTnI, high-sensitivity cardiac troponin I; BNP, B-type natriuretic peptide; NT-proBNP, N-terminal pro-B-type natriuretic peptide. ESS, effective sample size; IPW, inverse probability weighting; n, measured-cohort episode count; p, estimated probability of biomarker measurement.

Note: Low-probability counts use estimated probabilities before clipping. The highest-weight group comprised the top 1% of measured episodes, with the group size rounded upward to an integer. ESS/n (%) is 100 × ESS divided by the biomarker-measured count. Top 1% weight share is 100 × the sum of weights in the specified largest-weight group divided by the total measured-cohort weight. Lower-case p denotes the estimated measurement probability, not a statistical P value. The "all" and "measured" columns give episode counts in the complete cohort and measured subgroup, respectively.

Supplementary Table S6 — Panel C: Unclipped measurement-probability distributions

Biomarker	Measured status	n	P5	Median	P95
PCT	0	1868	0.2020	0.7081	0.8936
PCT	1	5858	0.5966	0.8493	0.9143
CK-MB	0	3118	0.0183	0.4420	0.8258
CK-MB	1	4608	0.3736	0.7670	0.8858
hs-cTnI	0	3054	0.0781	0.4529	0.8286
hs-cTnI	1	4672	0.3528	0.7663	0.8839
BNP	0	5959	0.0066	0.1902	0.4375
BNP	1	1767	0.1067	0.3172	0.5339
D-dimer	0	2939	0.0316	0.5387	0.8350
D-dimer	1	4787	0.4384	0.7553	0.8944
NT-proBNP	0	5285	0.0875	0.2958	0.5233
NT-proBNP	1	2441	0.1387	0.3880	0.6250

Abbreviations: PCT, procalcitonin; CK-MB, creatine kinase-MB; hs-cTnI, high-sensitivity cardiac troponin I; BNP, B-type natriuretic peptide; NT-proBNP, N-terminal pro-B-type natriuretic peptide. n, number of episodes; P1, 1st percentile; P5, 5th percentile; P95, 95th percentile; P99, 99th percentile.

Note: Measurement probabilities are unclipped; measured = 1 and unmeasured = 0. Distributions are shown in Figure S2. Full minima, maxima, and P1/P99 are provided in File S1 (propensity_overlap.csv). P5 and P95 denote the 5th and 95th percentiles. Each row uses its stated measured-status subgroup; n is the number of episodes in that subgroup.

Supplementary Table S7. Additional sensitivity and nonlinearity analyses — Panel A: D-dimer sensitivity analyses

Analysis	n	OR (95% CI)	P	ΔAUC
Exact-record candidates excluded	4786	0.890 (0.826–0.960)	0.002	0.00417
Sine/cosine month	4787	0.893 (0.828–0.962)	0.003	0.00506
Explicitly censored values excluded	4574	0.883 (0.813–0.959)	0.003	0.00402

Abbreviations: CRP, C-reactive protein; PCT, procalcitonin; CK-MB, creatine kinase-MB; hs-cTnI, high-sensitivity cardiac troponin I; BNP, B-type natriuretic peptide; NT-proBNP, N-terminal pro-B-type natriuretic peptide. OR, odds ratio; CI, confidence interval; AUC, area under the receiver operating characteristic curve; ΔAUC, added-model AUC minus base-model AUC. n, number of analyzed D-dimer-measured episodes; P, statistical P value.

Note: All models adjust for the observed variables; the seasonality model replaces 11 month indicators with sine and cosine terms. Exclusion analyses retain the same primary covariate specification. Here, n is the number of D-dimer-measured episodes included in each analysis. ORs describe viral positivity per doubling of D-dimer concentration and adjust for age, sex, requesting department, operational time, calendar month, and calendar year, with the stated seasonal substitution where applicable. ΔAUC compares the added D-dimer model with its base model in the same episodes. These analyses are post hoc and exploratory; exact-record matches are candidate duplicates, not confirmed duplicate specimens.

Supplementary Table S7 — Panel B: Biomarker concentration nonlinearity

Biomarker	LR χ^2	df	P
CRP	3.7026	2	0.1570
PCT	5.4075	2	0.0670
CK-MB	0.7497	2	0.6874
hs-cTnI	2.5647	2	0.2774
BNP	0.4846	2	0.7848
D-dimer	3.7743	2	0.1515
NT-proBNP	1.5672	2	0.4567

Abbreviations: CRP, C-reactive protein; PCT, procalcitonin; CK-MB, creatine kinase-MB; hs-cTnI, high-sensitivity cardiac troponin I; BNP, B-type natriuretic peptide; NT-proBNP, N-terminal pro-B-type natriuretic peptide. LR, likelihood ratio; χ^2, chi-square statistic; df, degrees of freedom; RCS, restricted cubic spline; log2, base-2 logarithm; P, statistical P value.

Note: The linear log2 concentration model was compared with a four-knot RCS model, with adjustment for age RCS and the other covariates. The two additional nonlinear terms give df = 2. Knots are the within-subset 5th, 35th, 65th, and 95th percentiles; exact values are reported in Panel E. Exploratory P values are not adjusted for multiplicity. LR χ^2 is the likelihood-ratio chi-square statistic for the additional nonlinear terms; df denotes degrees of freedom. These tests assess departure from a linear association on the log2 concentration scale.

Supplementary Table S7 — Panel C: Age nonlinearity

Cohort	LR χ^2	df	P	Linear-age AUC	Spline-age AUC
Complete cohort	9.5059	2	0.0086	0.8263	0.8274
CRP	9.6569	2	0.0080	0.8254	0.8265
PCT	0.1100	2	0.9465	0.7784	0.7786
CK-MB	0.4554	2	0.7963	0.7777	0.7780
hs-cTnI	1.5752	2	0.4549	0.7821	0.7825
BNP	3.1684	2	0.2051	0.7847	0.7854
D-dimer	0.1145	2	0.9444	0.7694	0.7698
NT-proBNP	2.5350	2	0.2815	0.7927	0.7931

Abbreviations: CRP, C-reactive protein; PCT, procalcitonin; CK-MB, creatine kinase-MB; hs-cTnI, high-sensitivity cardiac troponin I; BNP, B-type natriuretic peptide; NT-proBNP, N-terminal pro-B-type natriuretic peptide. AUC, area under the receiver operating characteristic curve; LR, likelihood ratio; χ^2, chi-square statistic; df, degrees of freedom; log2, base-2 logarithm; P, statistical P value.

Note: Age was modeled using four knots at 1, 65, 77, and 89 years, with two additional nonlinear terms. The complete-cohort comparison uses the base model. Biomarker-specific comparisons retain linear log2 concentration and all other covariates. AUC need not increase when likelihood increases. Tests are exploratory and not multiplicity-adjusted. Linear-age and spline-age AUCs refer to the corresponding models fitted to the same cohort. LR χ^2 tests the additional nonlinear age terms; df denotes degrees of freedom.

Supplementary Table S7 — Panel D: Alternative seasonal specification

Biomarker	n	OR (95% CI)	P	Base AUC	Extended AUC	ΔAUC
CRP	7639	0.985 (0.943–1.029)	0.502	0.8217	0.8217	−0.00007
PCT	5858	0.992 (0.955–1.031)	0.680	0.7701	0.7702	0.00010
CK-MB	4608	1.047 (0.974–1.126)	0.211	0.7679	0.7684	0.00059
hs-cTnI	4672	1.022 (0.976–1.070)	0.349	0.7724	0.7731	0.00074
BNP	1767	0.962 (0.870–1.064)	0.454	0.7721	0.7726	0.00047
D-dimer	4787	0.893 (0.828–0.962)	0.003	0.7544	0.7594	0.00506
NT-proBNP	2441	1.041 (0.975–1.111)	0.228	0.7841	0.7855	0.00140

Abbreviations: CRP, C-reactive protein; PCT, procalcitonin; CK-MB, creatine kinase-MB; hs-cTnI, high-sensitivity cardiac troponin I; BNP, B-type natriuretic peptide; NT-proBNP, N-terminal pro-B-type natriuretic peptide. OR, odds ratio; CI, confidence interval; AUC, area under the receiver operating characteristic curve; ΔAUC, extended-model AUC minus base-model AUC. n, number of measured episodes; log2, base-2 logarithm; P, statistical P value.

Note: Calendar month was replaced by $\sin(2\pi \times \text{month}/12)$ and $\cos(2\pi \times \text{month}/12)$, retaining age, sex, department, operational time, and categorical year. ORs are per doubling of concentration. These post hoc analyses are exploratory. The base model contains the stated covariates and seasonal terms; the extended model additionally includes log2 biomarker concentration. Both models use the same measured cohort. AUCs are apparent, and ΔAUC is calculated from unrounded values as extended-model AUC minus base-model AUC.

Supplementary Table S7 — Panel E: Concentration spline knots

Biomarker	5th percentile	35th percentile	65th percentile	95th percentile
CRP	−2.000000	2.435629	3.653060	4.812498
PCT	−4.321928	−2.599462	−0.739252	4.134015
CK-MB	−1.000000	0.584963	1.678072	4.153805
hs-cTnI	1.887525	3.678072	5.361066	10.387643
BNP	4.754888	6.672425	8.033423	11.008636
D-dimer	−1.089267	0.713696	1.769772	4.210233
NT-proBNP	6.139551	9.620220	11.847449	15.482619

Abbreviations: CRP, C-reactive protein; PCT, procalcitonin; CK-MB, creatine kinase-MB; hs-cTnI, high-sensitivity cardiac troponin I; BNP, B-type natriuretic peptide; NT-proBNP, N-terminal pro-B-type natriuretic peptide. log2, base-2 logarithm.

Note: Knots are on the log2 concentration scale within the corresponding measured cohort. These values define the tests in Panel B; age uses the fixed full-cohort knots reported in Panel C. Percentiles refer to the distribution of log2-transformed concentration within each biomarker-measured cohort; they are knot locations, not confidence limits or decision thresholds.

Supplementary Table S8. Associations between biomarker result availability and respiratory viral RT-PCR positivity.

Biomarker	Age specification	Episodes, n	Positive episodes, n	Adjusted OR (95% CI)	P value
PCT	Linear	7,726	983	0.702 (0.583–0.846)	<0.001
CK-MB	Linear	7,726	983	0.982 (0.805–1.199)	0.859
hs-cTnI	Linear	7,726	983	0.887 (0.735–1.069)	0.207
BNP	Linear	7,726	983	1.044 (0.849–1.282)	0.684
D-dimer	Linear	7,726	983	0.819 (0.672–0.999)	0.049
NT-proBNP	Linear	7,726	983	0.845 (0.698–1.023)	0.084
PCT	Restricted cubic spline	7,726	983	0.687 (0.570–0.828)	<0.001
D-dimer	Restricted cubic spline	7,726	983	0.829 (0.679–1.012)	0.066

Abbreviations: OR, odds ratio; CI, confidence interval; RT-PCR, reverse transcription polymerase chain reaction; PCT, procalcitonin; CK-MB, creatine kinase-MB; hs-cTnI, high-sensitivity cardiac troponin I; BNP, B-type natriuretic peptide; NT-proBNP, N-terminal pro-B-type natriuretic peptide; n, number of testing episodes.

Note: Each row represents a separate logistic regression model with respiratory viral positivity as the outcome and availability of a linked result for the specified biomarker as the exposure. The reference category is the absence of a linked result. Models include age, sex, requesting department, operational time, categorical calendar month, and calendar year. The spline models replace the linear age term with a restricted cubic spline with knots at 1, 65, 77, and 89 years. All rows use the full source cohort under the current primary outcome classification, rather than biomarker-measured subsets. Spline sensitivity results are reported for PCT and D-dimer only. These exploratory P values were not adjusted for multiple testing.

Supplementary Figure S1. Distribution of log2-transformed D-dimer concentration according to respiratory viral detection group.

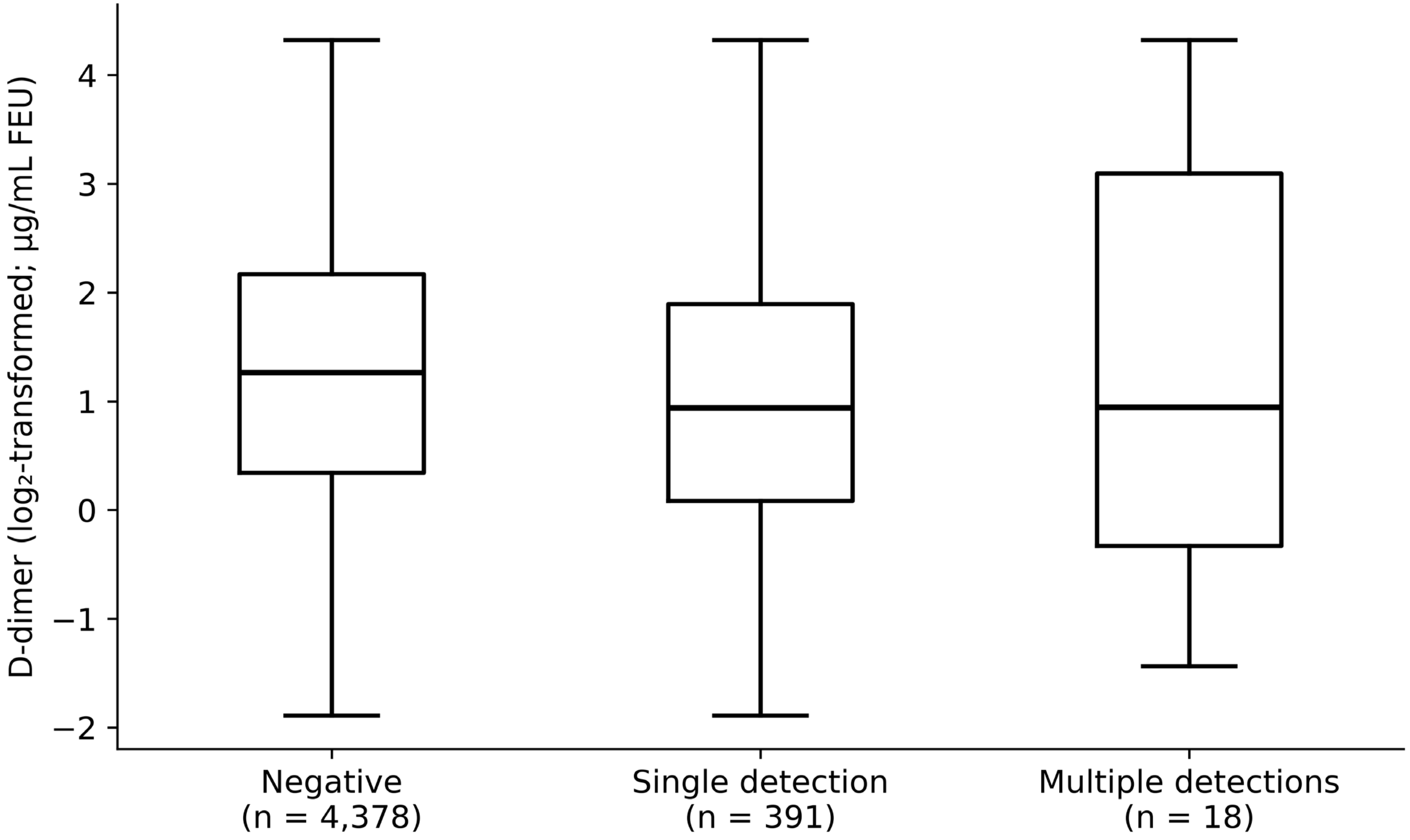

Note: Negative denotes no detected respiratory viral target (n = 4,378); single detection denotes one detected target (n = 391); multiple detections combine dual and three or more detected targets (n = 18). Boxes extend from the 25th to the 75th percentile of the log2-transformed values, center lines indicate medians, and whiskers extend to the minimum and maximum values used in the analysis. Concentrations were expressed in µg/mL FEU before log2 transformation. Values explicitly reported above the upper measurement limit were assigned that reported upper limit before transformation; thus, the upper whisker reflects the maximum analyzed value, not an uncensored maximum concentration. Group counts total 4,787 D-dimer-measured episodes.

Abbreviations: FEU, fibrinogen-equivalent units; log2, base-2 logarithm; n, number of testing episodes.

Supplementary Figure S2. Estimated probability of biomarker measurement among measured and unmeasured episodes

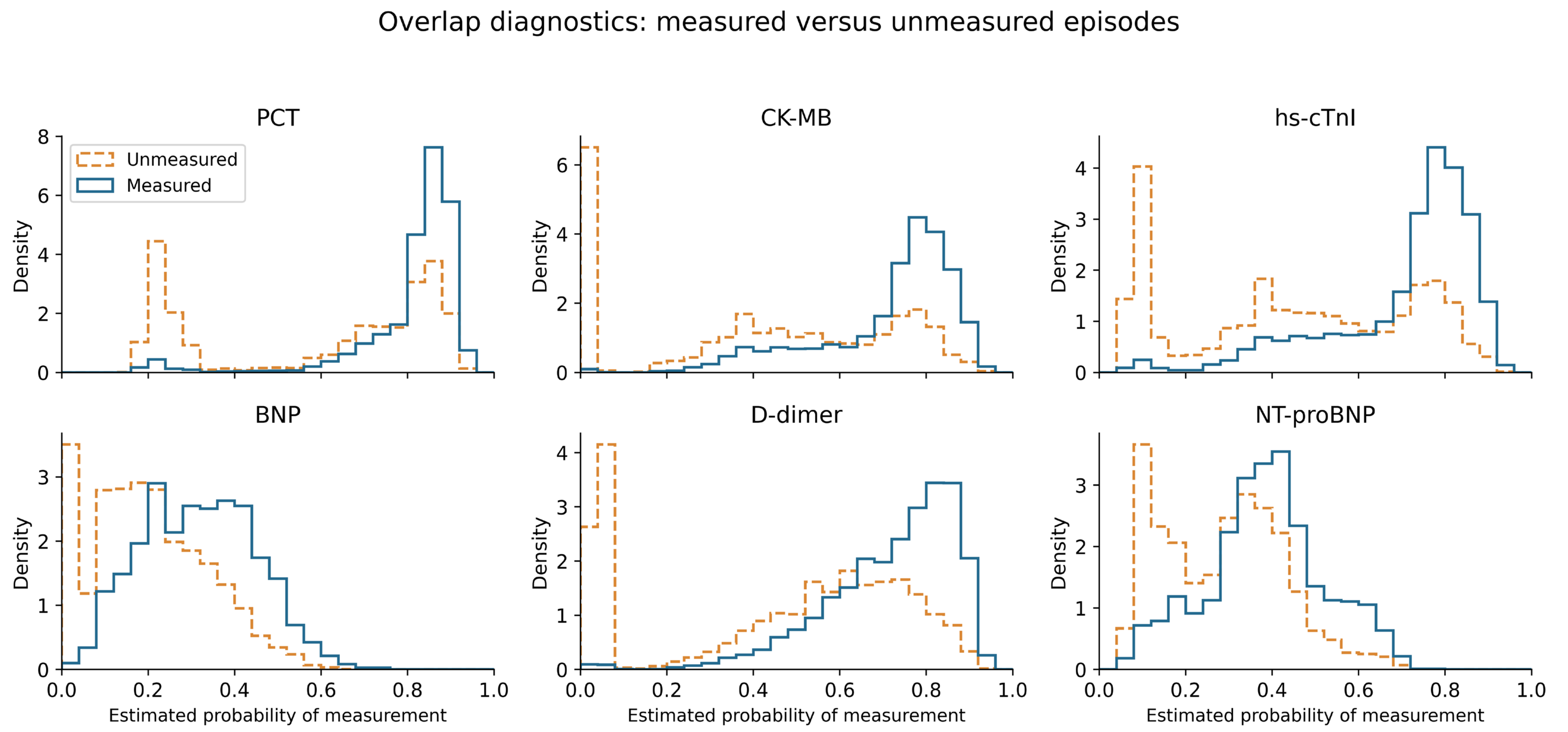

The histograms display density-normalized distributions of measurement probabilities before clipping, separately for each availability group. Probabilities were estimated from age, sex, requesting department, operational time, categorical calendar month, and categorical year. Each group's distribution is normalized separately; histogram height does not represent its sample size. CRP is not displayed because measurement was nearly universal and its measurement model had separation concerns. Limited distributional overlap may reduce the stability of IPW estimates; overlap on the estimated score does not establish exchangeability on unobserved clinical factors. Measured denotes a linked biomarker result in the released episode; unmeasured denotes no linked result. Absence of a linked result does not establish whether the test was ordered or performed. Measured episodes are shown with a solid line and unmeasured episodes with a dashed line.

Abbreviations: IPW, inverse probability weighting; CRP, C-reactive protein; PCT, procalcitonin; CK-MB, creatine kinase-MB; hs-cTnI, high-sensitivity cardiac troponin I; BNP, B-type natriuretic peptide; NT-proBNP, N-terminal pro-B-type natriuretic peptide.

Supplementary Figure S3. Stabilized inverse probability weight distributions among biomarker-measured episodes before percentile capping

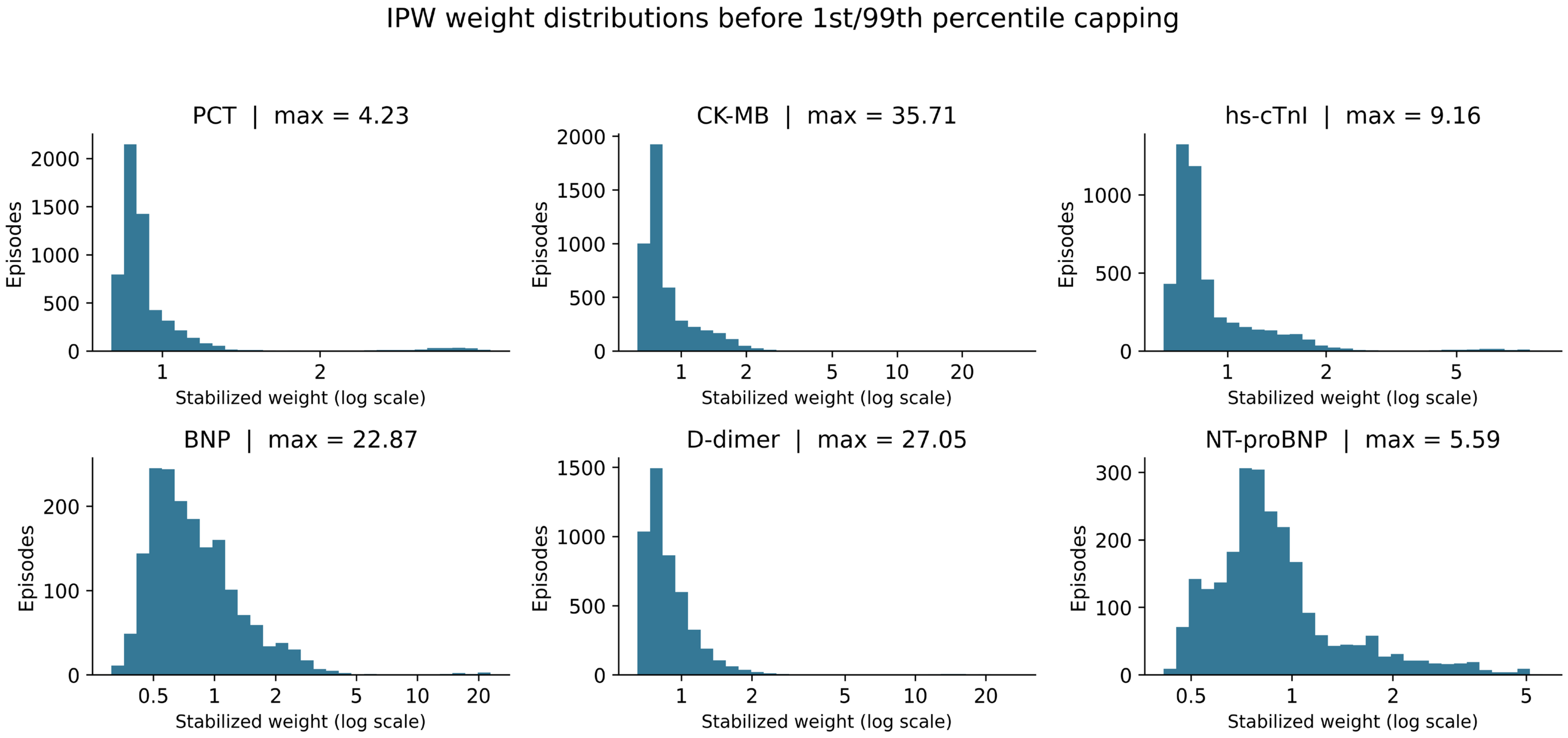

The x-axis is logarithmic and the y-axis reports episode counts; bins are spaced on the logarithmic scale. Weights equal the marginal measurement proportion divided by the estimated measurement probability, after clipping that probability to 0.01–0.99. The displayed distributions precede 1st/99th percentile weight capping. CRP is omitted because of near-universal measurement and separation concerns. Panel titles report maximum stabilized weights. The marginal measurement proportion is the biomarker-measured count divided by all 7,726 episodes; each histogram includes only the corresponding measured cohort.

Abbreviations: IPW, inverse probability weighting; max, maximum stabilized weight; CRP, C-reactive protein; PCT, procalcitonin; CK-MB, creatine kinase-MB; hs-cTnI, high-sensitivity cardiac troponin I; BNP, B-type natriuretic peptide; NT-proBNP, N-terminal pro-B-type natriuretic peptide.

Supplementary Figure S4. Adjusted associations with biomarker measurement — Panel a

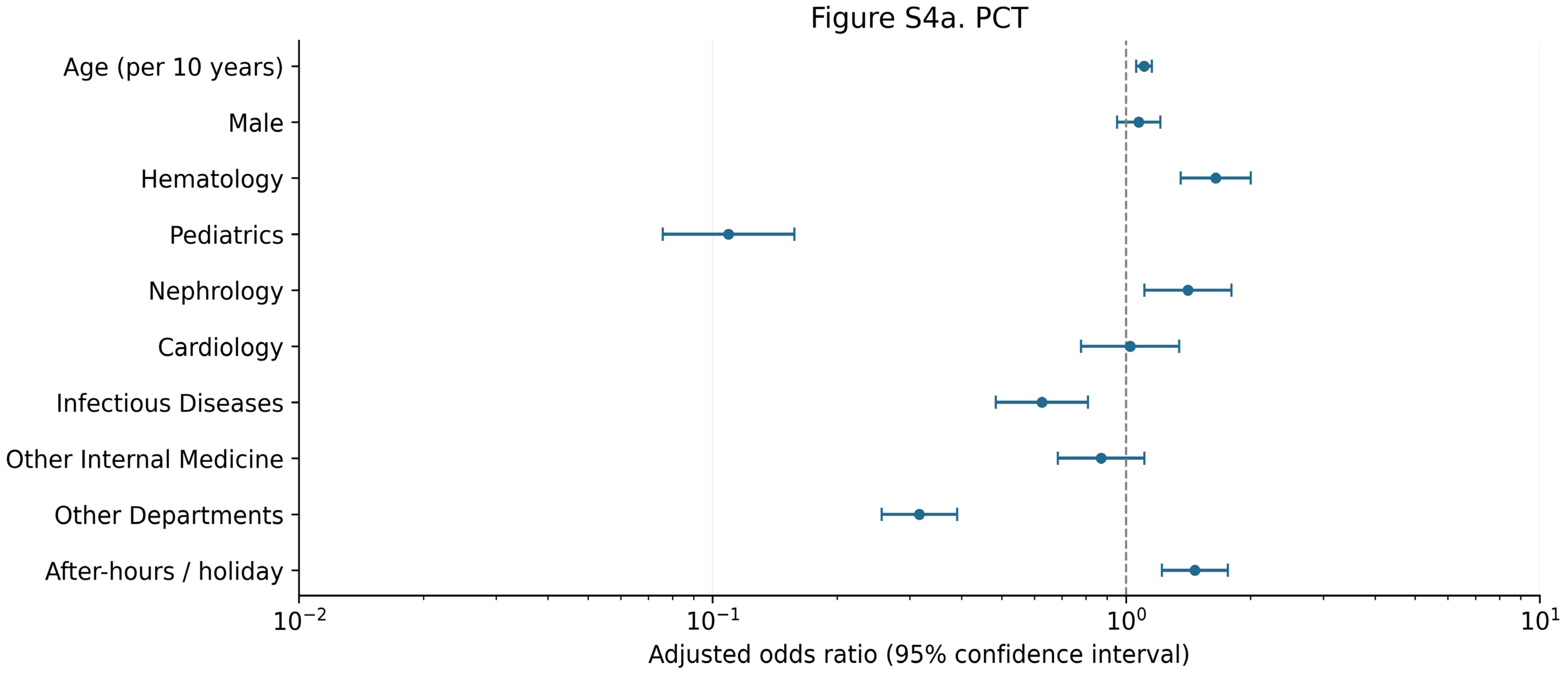

Supplementary Figure S4. Adjusted associations with biomarker measurement — Panel b

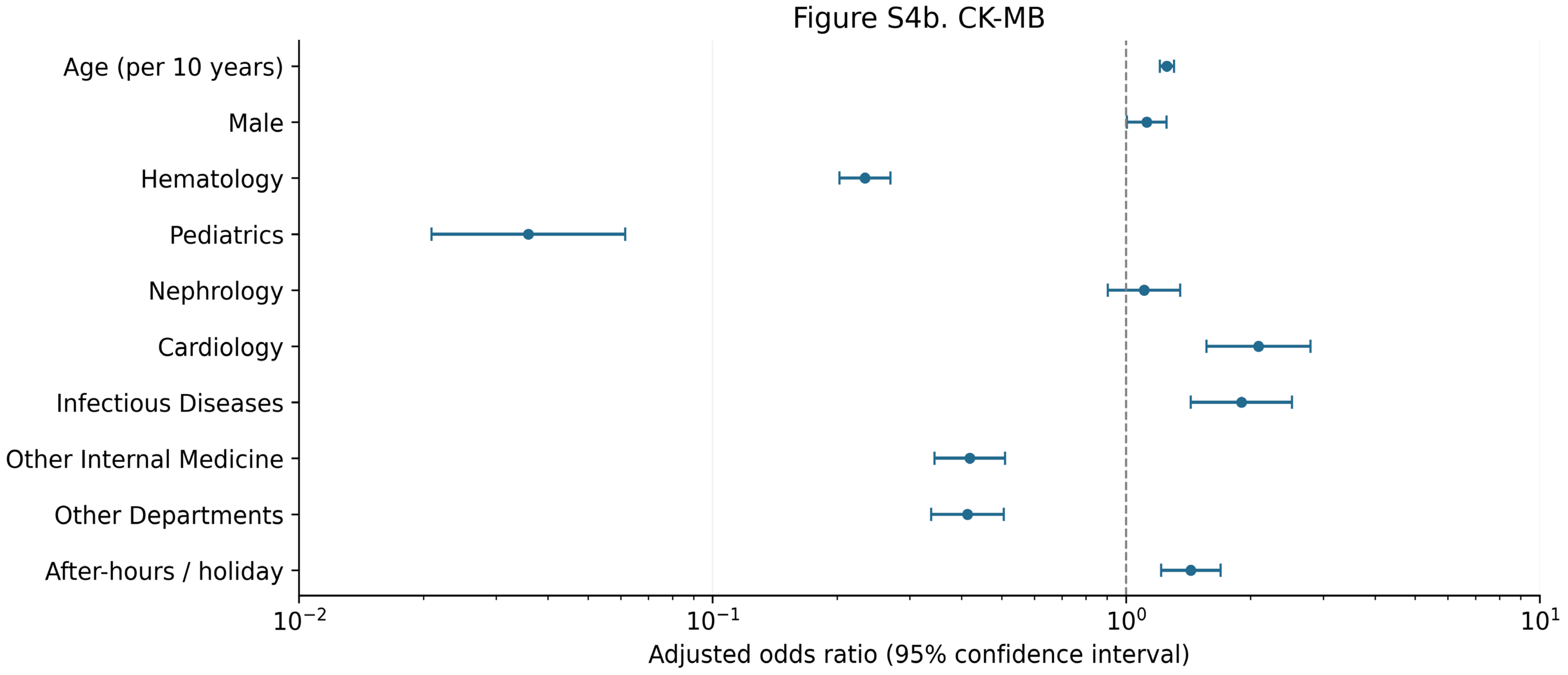

Supplementary Figure S4. Adjusted associations with biomarker measurement — Panel c

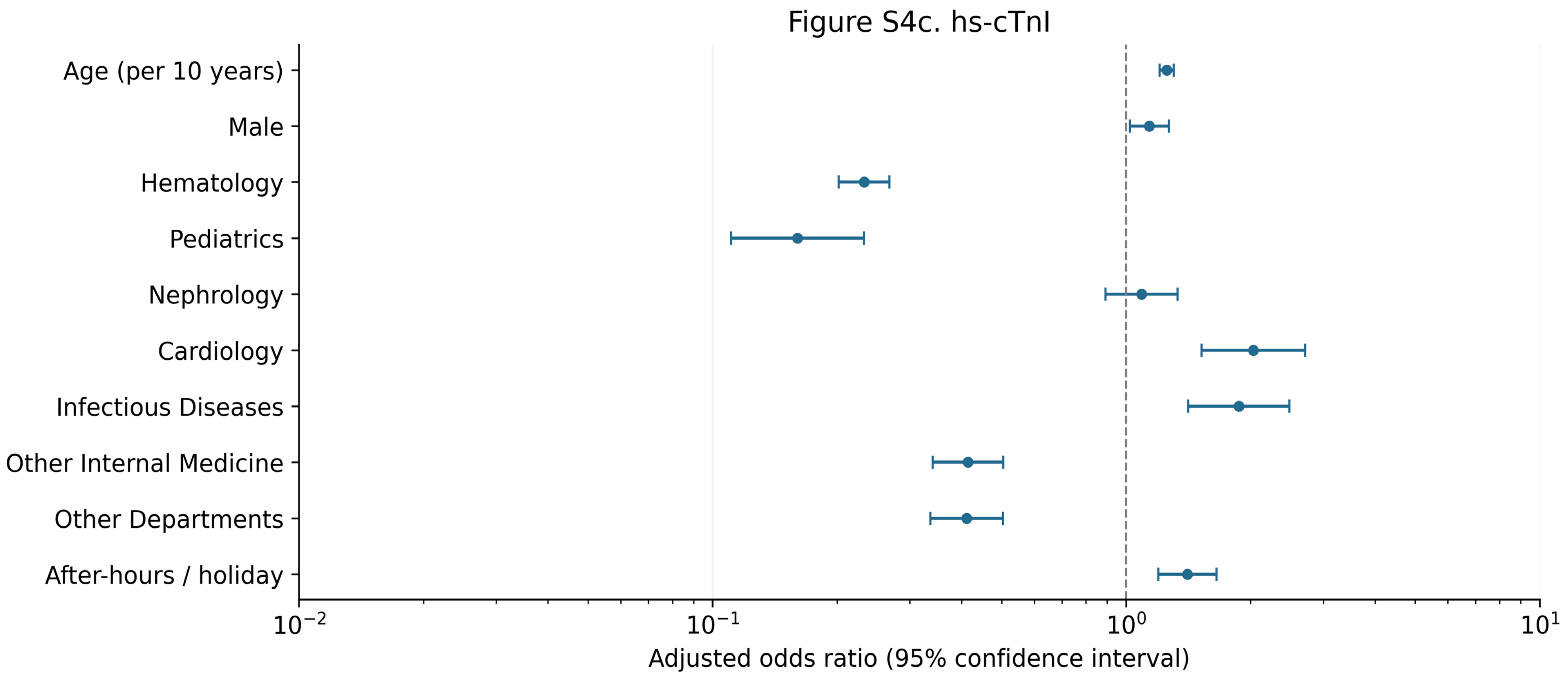

Supplementary Figure S4. Adjusted associations with biomarker measurement — Panel d

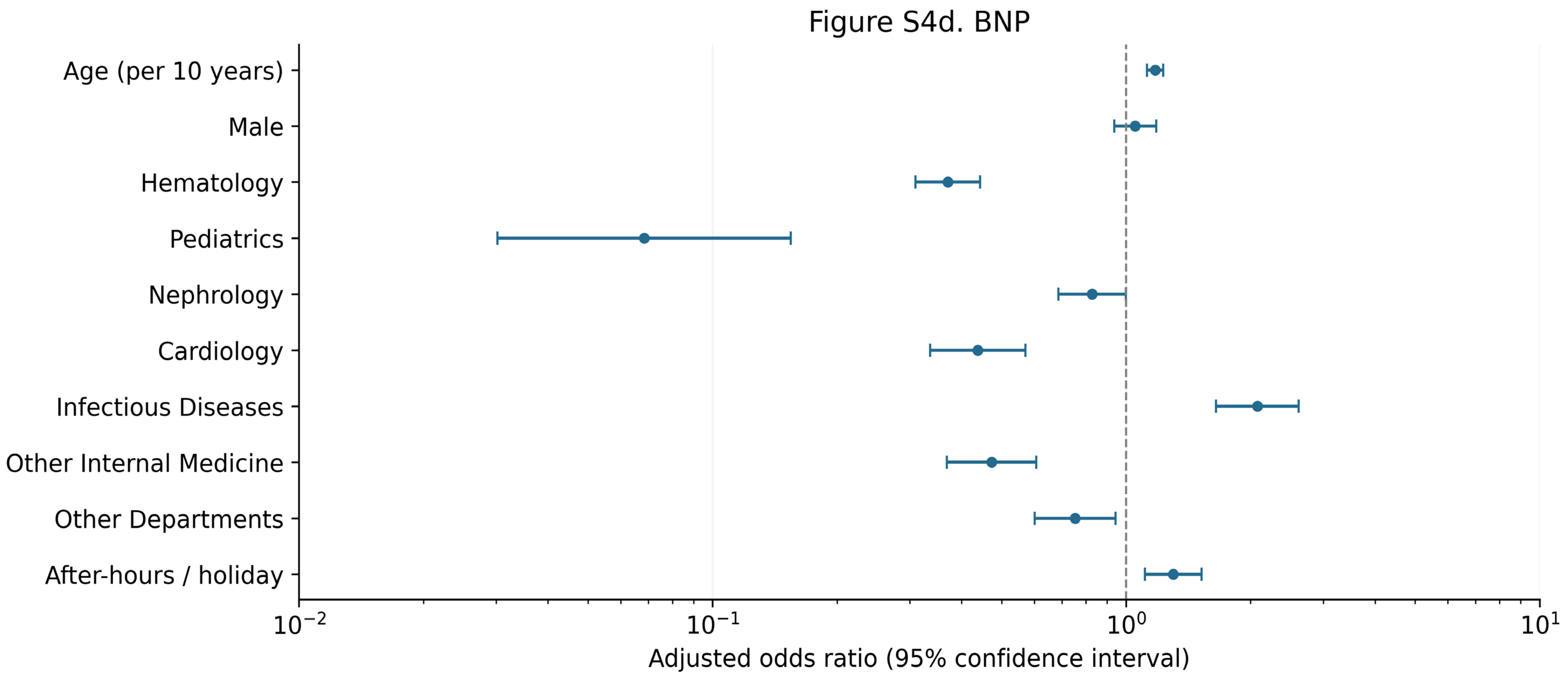

Supplementary Figure S4. Adjusted associations with biomarker measurement — Panel e

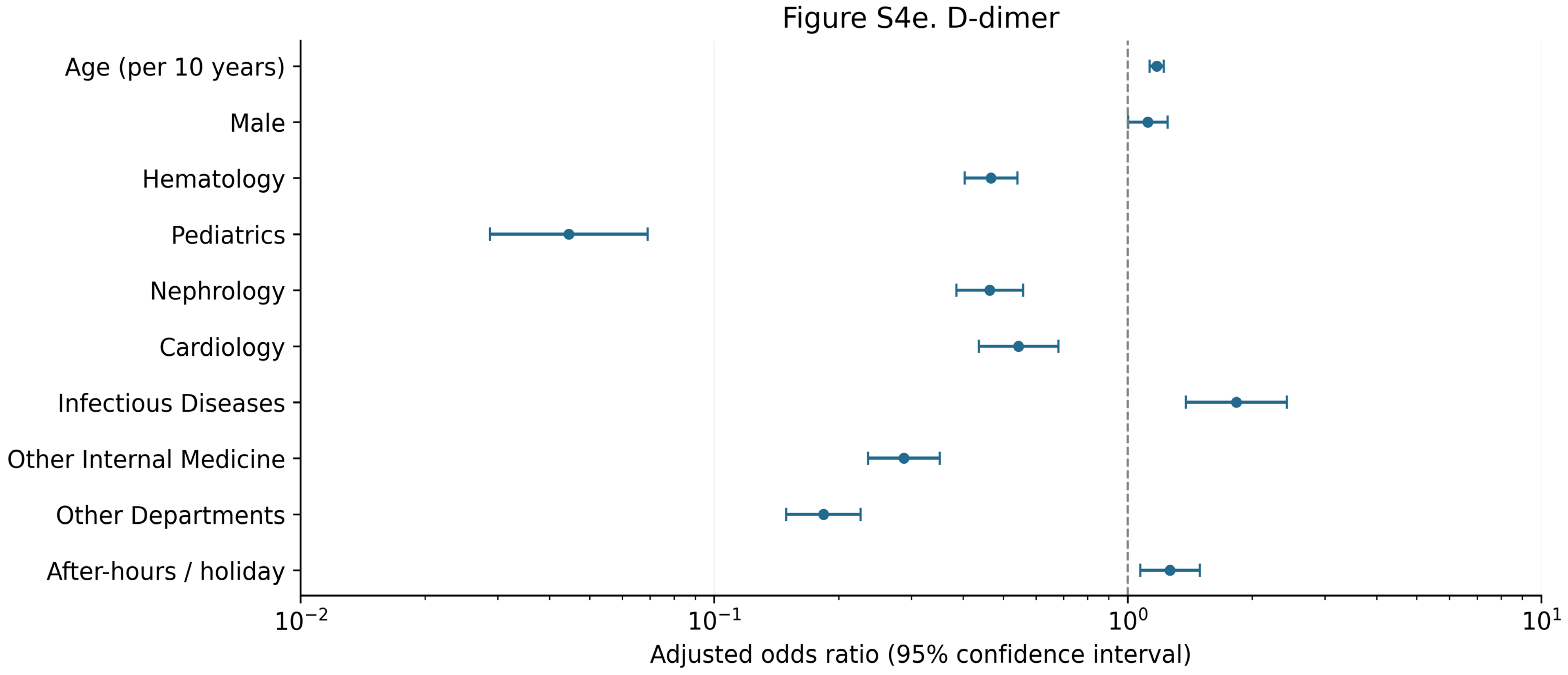

Supplementary Figure S4. Adjusted associations with biomarker measurement — Panel f

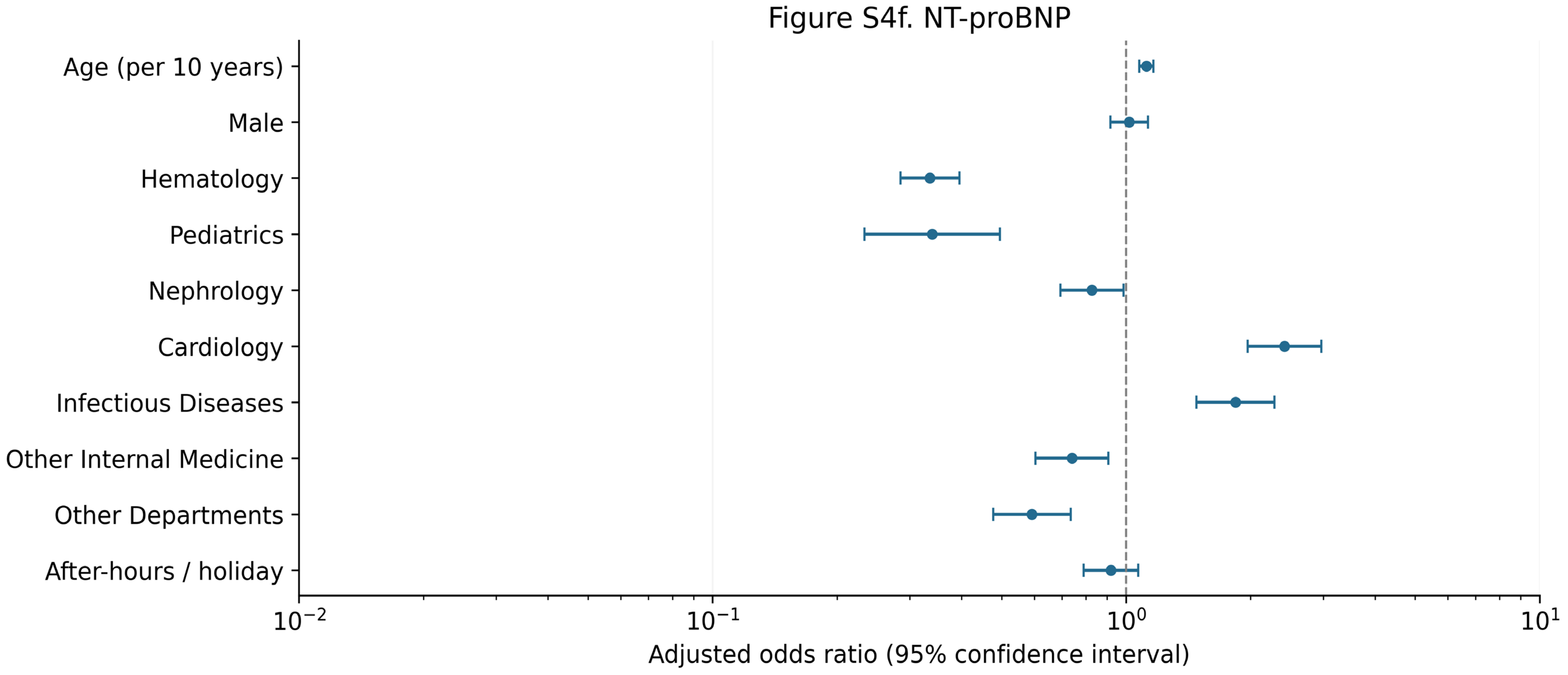

Panels a–f. Points denote ORs, and horizontal lines denote 95% confidence intervals from separate measurement-status models. Age is expressed per 10-year increase; reference categories are female, Pulmonology, and routine hours. All models also include categorical calendar month and year. CRP is omitted because measurement was nearly universal and stable estimation of the measurement model was limited by separation concerns. The outcome is biomarker measurement availability, not biomarker concentration or viral positivity. Points to the right of OR = 1 indicate higher adjusted odds of measurement relative to the stated reference; age is a continuous contrast per 10 years. The horizontal axis is logarithmic.

Abbreviations: OR, odds ratio; CI, confidence interval; CRP, C-reactive protein; PCT, procalcitonin; CK-MB, creatine kinase-MB; hs-cTnI, high-sensitivity cardiac troponin I; BNP, B-type natriuretic peptide; NT-proBNP, N-terminal pro-B-type natriuretic peptide.
